# Psychedelic-Assisted Interventions in Palliative Care: A Narrative Overview and Critical Evaluation

**DOI:** 10.3390/healthcare14111550

**Published:** 2026-06-02

**Authors:** Daniele Almeida Soares, Alessandro Gonçalves Campolina

**Affiliations:** 1 Johnson & Johnson Co., Ltd., Sao Paulo 04543-011, SP, Brazil; danifar007@gmail.com; 2 Faculdade de Medicina da Universidade de São Paulo (FMUSP), Universidade de São Paulo, Sao Paulo 01246-903, SP, Brazil; 3 Centro de Investigação Translacional em Oncologia, Instituto do Câncer do Estado de São Paulo, Faculdade de Medicina da Universidade de São Paulo (FMUSP), Universidade de São Paulo, Sao Paulo 01246-903, SP, Brazil

**Keywords:** psychedelics, palliative care, supportive care, psychedelic-assisted psychotherapy, cancer, chronic illness

## Abstract

**Background/Objectives**: Patients in palliative care frequently experience multidimensional suffering that extends beyond physical symptoms to include existential distress, demoralization, and loss of meaning. Psychedelic-assisted therapies (PAT), including ketamine-assisted psychotherapy (KAP), have re-emerged as promising interventions for these domains. This study aimed to provide a narrative overview and critical evaluation of the existing secondary literature on PAT in palliative care and serious illness and to examine the extent to which emerging best-practice recommendations are reflected in this literature. **Methods**: An overview of reviews with framework-based narrative synthesis was conducted, including narrative reviews, systematic reviews, scoping reviews, and meta-analyses addressing psychedelic-assisted interventions in patients with life-limiting illness. A comprehensive search of major databases was performed from inception to February 2026. Data extraction and narrative synthesis focused on clinical outcomes, safety, and the incorporation of key domains derived from recent interdisciplinary best-practice recommendations for PAT in palliative care. **Results**: Twenty-two reviews were included, synthesizing evidence primarily from early-phase clinical trials and observational studies, predominantly in oncology populations. Across reviews, PAT was consistently associated with reductions in depression, anxiety, and existential distress, along with improvements in quality of life and spiritual well-being. Safety profiles were generally favorable under controlled conditions. However, the incorporation of key therapeutic domains—such as preparation and integration, therapeutic setting, clinician training, and relational and biographical factors—was heterogeneous and often incomplete. Most reviews emphasized outcomes over process and context. **Conclusions**: The current body of secondary literature suggests potential application of PAT to address psychological and existential suffering in palliative care. However, the available evidence remains preliminary and is predominantly derived from small early-phase studies characterized by methodological heterogeneity, limited blinding, and highly selected populations. At the same time, the partial incorporation of emerging best-practice recommendations highlights a gap between evidence synthesis and normative clinical guidance.

## 1. Introduction

Palliative care emerged in response to the recognition that modern biomedicine, despite remarkable advances in disease-modifying treatments and life-prolonging technologies, has often struggled to adequately address the multidimensional suffering experienced by patients facing serious and life-limiting illness. Rooted in the modern hospice movement inspired by Dame Cicely Saunders, palliative care has emphasized a holistic, person-centered approach to care, encapsulated in the concept of total pain, which integrates physical, psychological, social, and spiritual dimensions of suffering [1,2]. This framework remains foundational to contemporary definitions of palliative care, including that of the World Health Organization, which explicitly recognizes spirituality and psychosocial well-being as integral components of quality end-of-life care [3].

Despite substantial progress in symptom control and supportive care, existential distress remains highly prevalent among patients with advanced illness. Manifestations such as demoralization, loss of meaning, death anxiety, spiritual anguish, and a desire for hastened death are commonly reported and are associated with diminished quality of life and increased psychological suffering [4,5]. Although several psychotherapeutic interventions—including dignity therapy, meaning-centered psychotherapy, CALM therapy, and supportive-expressive approaches—have demonstrated modest benefits, their effects are often limited in magnitude and duration, with inconsistent impact on depression and anxiety [5]. Pharmacological approaches, particularly antidepressants, have similarly shown limited efficacy in addressing existential distress per se, underscoring a persistent therapeutic gap in palliative care [4].

In this context, psychedelic-assisted interventions have re-emerged as a promising area of inquiry for addressing psychological and existential suffering associated with life-threatening illness. Psychedelics are “mind-manifesting” psychoactive substances capable of profoundly altering perception, affect, cognition, and sense of self [6]. When administered within structured psychotherapeutic frameworks emphasizing preparation, therapeutic support, and post-session integration, psychedelic-assisted therapy (PAT) may occasion experiences characterized by ego dissolution, enhanced meaning-making, emotional openness, and acceptance of mortality [7,8]. These experiential effects bear notable phenomenological similarities to states described in spiritual traditions and near-death experiences, positioning psychedelics as uniquely relevant to the existential challenges encountered at the end of life [8].

Historically, the intersection between psychedelics and palliative care is not novel. Early clinical studies conducted during the first wave of psychedelic research in the 1950s and 1960s—primarily involving lysergic acid diethylamide (LSD)—suggested reductions in pain, anxiety, and fear of death among patients with advanced cancer, alongside improvements in psychological well-being [4]. However, methodological limitations and the subsequent global prohibition of psychedelic substances in the late 1960s and early 1970s led to a prolonged interruption of this research. In recent years, a renewed “psychedelic renaissance” has produced a growing body of early-phase clinical trials investigating psilocybin-, LSD-, MDMA-, and ketamine-assisted interventions for depression, anxiety, and existential distress in patients with serious illness, particularly cancer [9,10].

Contemporary studies suggest that PAT may offer rapid and, in some cases, sustained reductions in anxiety, depressive symptoms, demoralization, and existential distress, along with improvements in quality of life and spiritual well-being [11,12]. Importantly, available data indicate a favorable safety profile in carefully selected patient populations, with no serious adverse events attributable to study drugs reported in recent trials [12]. Nonetheless, these findings must be interpreted cautiously. Existing trials have employed stringent inclusion and exclusion criteria, often enrolling highly motivated participants with relatively preserved prognoses, limited comorbidity, and, in some cases, prior psychedelic experience. Consequently, the generalizability of these results to patients with more advanced disease, complex medical conditions, or those receiving hospice care remains uncertain [9,12].

Beyond questions of efficacy and safety, psychedelic-assisted interventions raise broader ethical, epistemological, and clinical considerations. Similar to the historical development of hospice and palliative medicine itself, psychedelic therapy occupies a liminal space between biomedical treatment, psychotherapy, and spiritual care, drawing on humanistic psychology and experiential models of healing [13,14]. This convergence challenges conventional biomedical paradigms and invites renewed reflection on how medicine engages with meaning, suffering, and spirituality at the end of life.

In response to the rapid expansion of clinical interest and research activity in this field, an international interdisciplinary workshop recently convened clinicians, researchers, ethicists, and policymakers to formulate recommendations guiding best practice for psychedelic-assisted therapy (PAT) in the context of serious illness and palliative care [10]. These recommendations address key domains including patient selection, preparation and integration processes, safety and risk mitigation, therapeutic framing, clinician training and competencies, ethical governance, and alignment with core principles of palliative care. While this consensus effort represents an important step toward responsible clinical translation, it remains unclear to what extent these recommendations have been systematically incorporated into the secondary literature. Specifically, it is not yet known whether narrative, systematic, and scoping reviews that aim to synthesize the available evidence on psychedelic-assisted interventions in palliative care explicitly engage with or operationalize these best-practice recommendations.

The primary objective of this study is to provide a overview of reviews with framework-based narrative synthesis and critical evaluation of the existing secondary literature on psychedelic-assisted interventions in palliative care and serious illness. Specifically, this overview aimed to identify, synthesize, and appraise published narrative reviews, systematic reviews, and scoping reviews that summarize the current evidence on psychedelic-assisted therapy (PAT) for psychological, existential, and symptom-related outcomes in patients with life-limiting conditions.

A secondary objective is to examine the extent to which recently proposed best-practice recommendations for psychedelic-assisted therapy in palliative care—formulated through an international interdisciplinary workshop—are explicitly addressed, integrated, or operationalized within these reviews. By mapping the convergence and divergence between the synthesized evidence and emerging consensus-based guidance, this study sought to clarify how well current knowledge synthesis reflects evolving standards for ethical, clinical, and methodological best practice in this field.

## 2. Materials and Methods

### 2.1. Study Design

This study was designed as an overview of reviews with framework-based narrative synthesis, aiming to identify, appraise, and synthesize evidence from previously published reviews on psychedelic-assisted interventions in palliative care and serious illness. Given the conceptual, clinical, and methodological heterogeneity of the available literature, a framework-based narrative synthesis approach was adopted to critically examine how emerging best-practice dimensions are represented across the secondary literature, rather than to perform a strictly aggregative umbrella review focused solely on outcome synthesis.

This design was considered the most appropriate given the rapid expansion of the field and the growing number of secondary studies (including systematic, scoping, and narrative reviews) addressing overlapping but heterogeneous aspects of psychedelic-assisted therapies.

### 2.2. Eligibility Criteria

Reviews were eligible for inclusion if they met the following criteria: (1) were narrative reviews, systematic reviews, scoping reviews, or meta-analyses; (2) addressed the use of psychedelic substances—including classic serotonergic psychedelics (e.g., psilocybin, lysergic acid diethylamide [LSD], mescaline, and dimethyltryptamine [DMT]/ayahuasca), entactogens (e.g., 3,4-methylenedioxymethamphetamine [MDMA]), dissociative agents (e.g., racemic ketamine and esketamine), and related compounds such as ibogaine—administered within a therapeutic or clinical framework; (3) focused on patients with serious, life-limiting, or advanced illness, including palliative care, hospice care, or oncology populations; and (4) reported outcomes related to psychological distress, existential suffering, quality of life, spiritual well-being, symptom burden, or end-of-life experiences.

Reviews were excluded if they focused exclusively on recreational use, neurobiological mechanisms without clinical application, or psychiatric conditions unrelated to serious or life-limiting illness. Studies conducted exclusively in healthy populations were also excluded. Primary studies, including clinical trials, standalone qualitative studies, and case reports, were not considered eligible. Additionally, commentaries, editorials, and letters to the editor were excluded, as were review protocols without reported results. Reviews lacking a minimal description of their methodological approach were also excluded. Consensus statements and other non-review publications were excluded unless embedded within a broader review article.

### 2.3. Information Sources and Search Strategy

A comprehensive literature search was conducted in PubMed/MEDLINE, Scopus, Web of Science, Embase (via Ovid), and LILACS from database inception to 2 February 2026.

The search strategy was developed based on key components of the research question, focusing on population (patients with serious illness or receiving palliative care) and intervention (psychedelic-assisted therapies).

To ensure comprehensive identification of relevant interventions, search strategies were structured separately for each psychedelic compound (psilocybin, ketamine, esketamine, lysergic acid diethylamide [LSD], N,N-dimethyltryptamine [DMT], mescaline, 3,4-methylenedioxymethamphetamine [MDMA], and ibogaine), incorporating both controlled vocabulary (e.g., MeSH in MEDLINE and Emtree in Embase) and free-text terms. These intervention-specific terms were combined with terms related to serious illness and palliative care (e.g., “palliative care”, “hospice care”, “end-of-life care”, “terminal care”, “cancer”, “neoplasms”).

For the LILACS database, search terms were additionally translated into Portuguese and Spanish to ensure comprehensive retrieval of regional literature. Search strategies were adapted to the syntax and indexing systems of each database, including the use of subject headings, text words, and database-specific search operators.

No study design filters were applied during the search phase in order to maximize sensitivity. Eligibility for inclusion as review articles was determined during the study selection process based on predefined inclusion criteria.

For PRISMA reporting, results from all compound-specific searches were combined within each database to generate the final number of identified records.

The full search strategy for each database is provided in the Supplementary Materials (Table S1). Reference lists of included reviews were also hand-searched to identify additional eligible publications.

### 2.4. Study Selection

All retrieved records were screened independently by two reviewers at the title and abstract level. Full-text articles were subsequently assessed for eligibility based on predefined inclusion and exclusion criteria. Disagreements were resolved through discussion and consensus. A list of excluded studies at the full-text stage, along with reasons for exclusion, is provided in the Supplementary Materials (Table S2).

**Figure 1:**
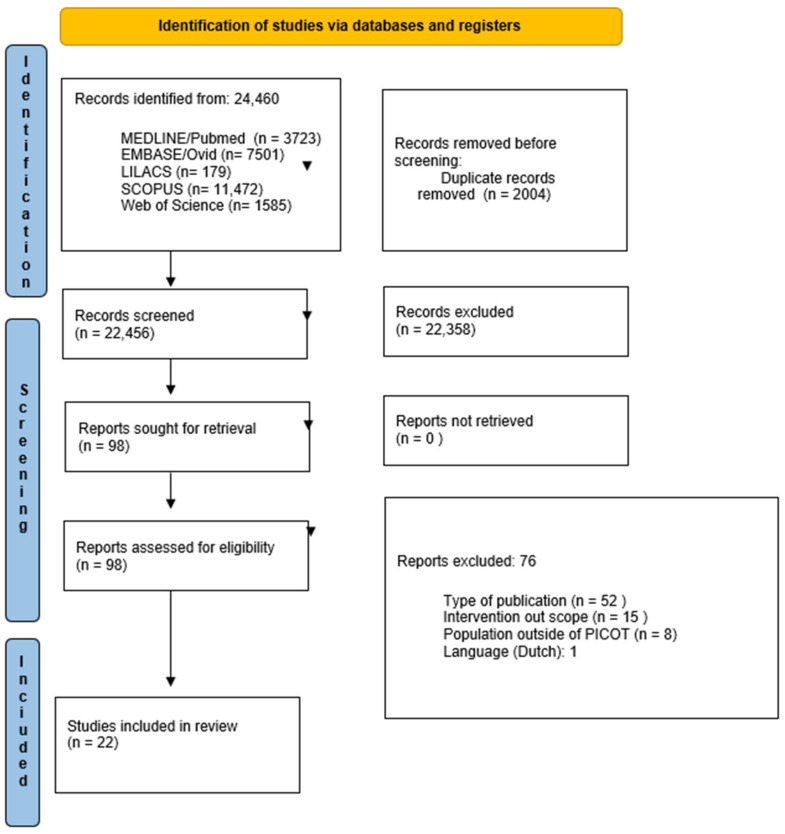
PRISMA flow diagram of the study selection process. Own elaboration, flow adapted from Page MJ, McKenzie JE, Bossuyt PM, Boutron I, Hoffmann TC, Mulrow CD et al. The PRISMA 2020 statement: an updated guideline for reporting systematic reviews. https://doi.org/10.1136/bmj.n7. [15].

The study selection process is summarized using a PRISMA flow diagram [15] adapted for overviews of reviews (Figure 1).

### 2.5. Data Extraction

Data were extracted independently by two reviewers using a standardized data extraction form. Extracted variables included author, year, type of review, psychedelics addressed, population or clinical context, number of included studies, outcomes analyzed, principal findings reported, safety considerations, relevance for palliative care, reported methodological limitations, and a critical analysis conducted by the reviewers.

In addition, each review was examined for explicit consideration of key domains relevant to psychedelic-assisted therapy in palliative care, including length of therapy, important clinical indications, intrinsic motivation, mystical-type experiences, integration with the palliative care healthcare model, personal or family issues, clinical training, biographical and relational considerations, and dosing and administration.

### 2.6. Synthesis and Analytical Framework

A narrative synthesis was undertaken to compare and contrast findings across reviews. Particular attention was given to identifying areas of convergence, divergence, and omission in the secondary literature. The recommendations emerging from the international interdisciplinary workshop on psychedelic-assisted therapy in palliative care were used as an interpretive framework to assess whether and how existing reviews engage with best-practice considerations beyond efficacy and safety alone. Given the qualitative and heterogeneous nature of the included literature, no quantitative meta-analysis was performed.

Each included review was independently coded according to the extent to which predefined framework domains were addressed within the publication. The analytical domains were derived from recent international interdisciplinary consensus recommendations concerning psychedelic-assisted therapy (PAT) in palliative care [ref. framework] and were operationalized through a structured codebook developed for this review. Detailed operational definitions for each domain are provided in the Supplementary Materials (Table S3). Coding classifications were organized into three categories: (1) “Explicit”, defined as domains that were clearly and substantively discussed as relevant components of care delivery, therapeutic process, or intervention design, according to the definition of the domain; (2) “Partial”, defined as domains that were briefly mentioned or indirectly referenced without structured or detailed discussion; and (3) “Absent”, defined as domains that were not identified within the review. Independent coding was conducted by two reviewers, and disagreements were resolved through consensus discussion to improve analytical consistency and transparency.

## 3. Results

A total of 22 studies were included in this overview, all of which were considered eligible according to the previously defined criteria. These studies comprised narrative reviews, systematic reviews, scoping reviews, and meta-analyses addressing psychedelic-assisted interventions in palliative care and in populations with serious and potentially life-limiting illnesses.

Table 1 presents a summary of the main characteristics of the included studies, including authors, year of publication, review type, psychedelics addressed, population or clinical context, number of included studies, and outcomes analyzed. Detailed information on additional variables, including main findings, safety, relevance to palliative care, and reported methodological limitations, is provided in the Supplementary Materials (Table S4).

Regarding the psychedelics addressed, most reviews focused on psilocybin, either alone or in combination with other classic psychedelics such as LSD and ayahuasca (DMT/MAOIs). A relevant subset of studies also included MDMA and ketamine or esketamine, either as atypical psychedelic interventions or within the context of dissociative-assisted psychotherapy. Several reviews adopted a cross-cutting approach, analyzing more than one substance, while others focused on a single compound or a specific therapeutic model.

The clinical outcomes most frequently examined included depression, anxiety, existential distress, quality of life, spiritual well-being, demoralization, and attitudes toward death. In many studies, these outcomes were discussed jointly, reflecting a multidimensional understanding of suffering in the context of serious illness. Issues related to safety and tolerability were also addressed in the majority of reviews, although generally in a descriptive manner, based on reports from the primary studies included.

When mapping the content of the reviews according to the analytical framework adopted in this study, the incorporation of the different domains proved to be heterogeneous. Domains such as clinical indications, psychological and existential outcomes, and general description of the intervention model were widely addressed in most reviews. Conversely, other core elements of the framework—such as length of therapy, preparation and integration processes, mystical-type experiences, integration with palliative care models, family and relational issues, clinical training and competencies, and ethical and organizational aspects—were addressed variably and, in many cases, only partially or were absent (Table 2). Table 2 presents the domain-level classification for each included study according to the extent of coverage, categorized as “Explicit”, “Partial”, or “Absent”. Detailed data extraction supporting these classifications is provided in the Supplementary Materials (Table S5).

Overall, relatively few reviews explicitly operationalized these domains as formal analytical criteria. Instead, many elements of the framework appeared vaguely throughout the narratives, without systematic structure or comparative evaluation across the reviewed studies. This variability limited direct comparison between reviews regarding the degree of adherence to emerging recommendations for the practice of psychedelic-assisted therapies in palliative care.

**Figure 2:**
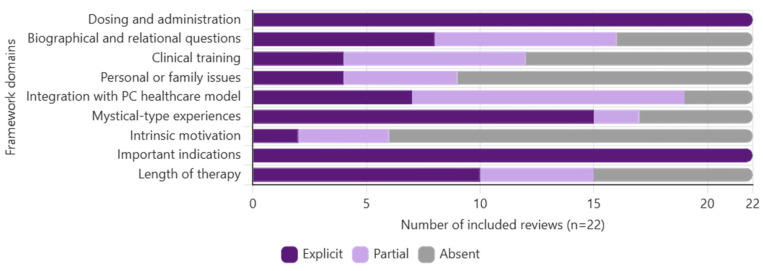
Framework domain coverage across included reviews.

Figure 2 graphically illustrates the frequency with which the included studies addressed each of the framework domains, highlighting how many reviews explicitly, partially, or did not address each component. Accordingly, domains related to clinical indications and outcomes were the most frequently reported, whereas aspects such as integration within palliative care systems, professional training, biographical dynamics, and family issues were less consistently addressed.

Taken together, the results indicate that the 22 included reviews report preliminary evidence regarding psychedelic-assisted interventions in contexts of serious illness, with consistent signals of potential clinical benefit. Nevertheless, the framework-based analysis reveals a partial and uneven incorporation of components considered essential for responsible clinical practice, underscoring the importance of analyses that extend beyond efficacy and safety to also encompass processes, context, and integration with the principles of palliative care.

## 4. Discussion

This overview of reviews with framework-based narrative synthesis sought to synthesize the current state of secondary evidence on psychedelic-assisted interventions in palliative care, while examining the extent to which this body of literature engages with emerging best-practice recommendations. Overall, the findings suggest a field characterized by promising but methodologically constrained evidence, alongside a partial and uneven incorporation of normative clinical frameworks.

Across the included reviews, there is a relatively consistent signal that psychedelic-assisted therapies—particularly those involving psilocybin—may contribute to meaningful reductions in depression, anxiety, and existential distress among patients with serious illness. Improvements in spiritual well-being, sense of meaning, and death acceptance were also frequently reported, often in association with the intensity or significance of subjective or “mystical-type” experiences. These findings align with earlier primary studies suggesting that the therapeutic effects of psychedelics may be mediated less by direct symptom suppression and more by transformative experiential processes [36,37,38,39,40,41].

Importantly, these benefits were not limited to narrowly defined psychiatric outcomes but extended to broader domains central to palliative care, including quality of life, psychosocial functioning, and relational dimensions of suffering. Such multidimensional effects resonate with the concept of “total pain” [42] and seem to support the hypothesis that psychedelic-assisted interventions may uniquely address the intertwined psychological, existential, and spiritual dimensions of serious illness [43,44].

However, it is equally important to emphasize that these findings derive largely from small, highly controlled clinical trials, often conducted in specialized research environments with carefully selected participants. As such, the observed therapeutic signal should be interpreted as preliminary and context-dependent, rather than definitive evidence of effectiveness in real-world palliative care settings.

The overview highlights several well-recognized but still unresolved methodological challenges in psychedelic research [9,45]. First, functional unblinding remains a pervasive issue, as the subjective effects of psychedelics make it difficult to maintain masking, potentially inflating expectancy effects and outcome estimates [46]. Second, the consistent use of multicomponent interventions—combining pharmacological administration with structured preparation, therapeutic support, and integration—renders it difficult to disentangle the specific contribution of the psychedelic compound from that of the surrounding psychotherapeutic and environmental context [47].

In addition, the evidence base is marked by substantial heterogeneity in dosing regimens, number of sessions, therapeutic models, and outcome measures, limiting comparability across studies and complicating efforts at synthesis. The absence of large-scale, multicenter phase III trials further constrains the robustness of current conclusions, while the lack of head-to-head comparisons between different compounds or active comparators leaves important clinical questions unanswered.

A particularly salient limitation concerns the limited representativeness of study populations. Most trials have been conducted in high-income countries, often with predominantly white, relatively well-resourced participants. This raises concerns regarding the generalizability and equity of findings, especially in the context of palliative care, where patients frequently present with multimorbidity, frailty, and complex psychosocial needs. Data on drug–drug interactions, long-term safety, and outcomes in more medically vulnerable populations remain notably scarce [48,49,50].

A central aim of this overview was to examine whether existing reviews engage with the emerging consensus on best practices for psychedelic-assisted therapy, as articulated in recent international workshop recommendations. The analysis suggests that while many reviews implicitly acknowledge key elements—such as the importance of preparation, therapeutic support, and integration—there is limited systematic engagement with these domains as structured evaluative frameworks [16,28,30,31,32,33,35].

Core components emphasized by the workshop, including patient selection criteria, clinician training and competencies, ethical safeguards, and integration within palliative care systems, are often addressed only superficially or inconsistently in the review literature [18,19,20,21,22,23,24,25,26,27,28,29,30,31,32,33,34,35]. In particular, domains such as ethical governance, management of patient vulnerability, and alignment with palliative care principles remain underdeveloped in many syntheses, which tend to focus more narrowly on efficacy outcomes [16,28,32,33].

Moreover, few reviews explicitly conceptualize psychedelic-assisted therapy as a complex intervention embedded within a broader care model, as opposed to a discrete pharmacological treatment [19,20,21,32]. This conceptual gap may contribute to disproportionate emphasis on outcome metrics at the expense of process variables, such as therapeutic alliance, experiential depth, and integration practices, which are increasingly recognized as central to clinical effectiveness.

Taken together, these findings suggest that psychedelic-assisted interventions in palliative care should be understood not simply as novel therapeutics, but as multidimensional care processes that intersect with foundational questions of meaning, identity, and relationality at the end of life. This positions PAT within a domain that is both clinically promising and ethically complex, requiring careful integration into existing models of care.

From a clinical perspective, the preliminary evidence indicates a signal of benefit regarding the potential of these interventions to address otherwise refractory forms of suffering, particularly those related to existential distress. However, this potential must be balanced against the current limitations of the evidence base and the need for robust safeguards in vulnerable populations [9,23].

From a conceptual perspective, the field may benefit from moving beyond narrowly reductive efficacy paradigms toward frameworks that account for context, embodiment, and relational dynamics. Such an approach would be more consistent with both palliative care philosophy and contemporary models of psychedelic therapy, which emphasize the co-constitutive roles of drug, set, and setting [51].

Future research should prioritize: large, multicenter trials with more diverse and clinically representative populations; greater standardization of intervention protocols and outcome measures; explicit investigation of mechanisms of action, including the role of subjective experience and therapeutic context; systematic evaluation of safety, drug interactions, and long-term outcomes in palliative populations.

In parallel, there is a need for greater integration of normative frameworks, such as those proposed by international expert consensus, into both primary studies and evidence syntheses. This includes developing operational criteria for best practice, which can guide not only clinical implementation but also the design and evaluation of future research.

From an implementation science perspective, future research on psychedelic-assisted therapy (PAT) in palliative care may benefit from conceptualizing these interventions not merely as pharmacological treatments, but as complex, multicomponent models of care delivery. Similar to care bundles and structured clinical pathways developed in other high-risk healthcare settings, PAT involves the coordinated interaction of multiple interdependent elements, including therapeutic setting, preparatory and integration processes, multidisciplinary team coordination, clinician competencies, safety monitoring, and ethical governance structures [10]. Recent literature on care bundle development in clinical risk management has emphasized the importance of evidence-based pathway design, multidisciplinary consensus-building, implementation feasibility, fidelity monitoring, and continuous evaluation mechanisms as core dimensions of clinically governable interventions [52]. In this context, implementation science frameworks such as the Consolidated Framework for Implementation Research (CFIR) may offer useful conceptual tools for assessing implementation readiness, organizational preparedness, workforce training requirements, institutional culture, and barriers to sustainable integration of PAT within palliative care systems [53]. Such an approach may be particularly relevant given that psychedelic-assisted interventions require competency-based delivery models, interdisciplinary collaboration, integration of spiritual and psychosocial dimensions of care, continuity across preparation-treatment-integration phases, and clear referral and follow-up systems [10]. Accordingly, future translational research should move beyond efficacy-oriented paradigms alone and also investigate how PAT can be operationalized through reproducible, ethically accountable, and clinically monitorable models of care within real-world palliative care settings.

This overview is subject to limitations inherent to narrative syntheses of secondary literature. Although systematic search strategies were employed, the analysis remains dependent on the scope and quality of existing reviews, which themselves vary in methodological rigor. Furthermore, given the substantial overlap of primary studies across reviews, findings should not be interpreted as independent replication. In addition, the interpretation of alignment with workshop recommendations necessarily involves a degree of conceptual judgment, as these recommendations are not always operationalized in ways that lend themselves to straightforward comparison.

## 5. Conclusions

The current body of review literature on psychedelic-assisted interventions in palliative care provides encouraging but incomplete evidence of therapeutic potential. While clinical signals of efficacy seem to be supported across multiple domains of suffering, they are tempered by significant methodological and contextual limitations. At the same time, the partial incorporation of emerging best-practice recommendations highlights a gap between evidence synthesis and normative clinical guidance.

Although the literature reviewed suggests preliminary therapeutic potential under highly controlled research 22 conditions, current evidence remains insufficient to support broad clinical implementation. Bridging this gap will be essential for the responsible development of the field.

## Figures and Tables

**Table 1:** General characteristics of the included studies.

Author, Year	Review Type	Psychedelics Addressed	Population/Clinical Context	Number of Included Studies	Outcomes Analyzed
Maia et al., 2022 [11]	Systematic review	LSD, psilocybin, DPT, ketamine and MDMA	Patients with life-limiting conditions, advanced or terminal illness, predominantly cancer	20 studies	Control of physical, psychological, social, and spiritual symptoms
Belitzky et al., 2025 [16]	Narrative Review	LSD, psilocybin, mescaline, and DMT	Patients with cancer	23 studies	Cancer pain and associated psychological distress
Reiche et al., 2018 [17]	Systematic review	LSD, psilocybin and DPT	Patients with life-limiting conditions, advanced or terminal illness	11 studies	Anxiety, depression, existential distress, quality of life, fear of death, psychological outcomes
Schuman, et al., 2025 [18]	Systematic review and meta-analysis	Psilocybin, ketamine, MDMA and LSD	Patients with cancer or survivors with depression, anxiety, demoralization, and existential distress	15 studies	Depression, anxiety, suicidal ideation and existential distress
Marchi, et al., 2024 [19]	Systematic review and network meta-analysis	Psilocybin, ketamine, MDMA and LSD	Patients with terminal illness, such as advanced cancer	9 studies	Post-treatment measures of depression and anxiety
Barbosa et al., 2023 [20]	Systematic review	Racemic ketamine and S-ketamine (esketamine)	Patients with life-threatening illnesses	32 studies	Depressive symptom outcomes
Jing, Hoeh & Menkes, 2022 [9]	Scoping review	Ketamine, psilocybin, MDMA and LSD	Patients with life-threatening illnesses receiving palliative or hospice care	25 studies	Depression, anxiety, and existential distress at end of life.
Schimmers et al., 2022 [21]	Systematic review	Psilocybin, LSD, MDMA and ketamine	Patients with a terminal illness	33 studies	Depression, anxiety, and existential distress
Yu et al., 2021 [22]	A systematic review and meta-analysis	Psilocybin	Patients with life-threatening conditions, such as cancer and HIV	5 studies	Anxiety symptoms, including state anxiety and trait anxiety
Schipper et al., 2024 [23]	Systematic Review	Psilocybin, LSD and MDMA	Patients with life-threatening diseases	6 studies	Anxiety, depression and existential distress
White et al., 2023 [24]	Comprehensive literature review	Psilocybin, LSD and MDMA	Patients with cancer	5 studies	Anxiety and depression
Lapid et al., 2025 [25]	Systematic review	Psilocybin/ psilocybin-assisted therapy	Patients with cancer with psychological and existential distress	14 studies	Depression, anxiety and existential distress
Stephen Ross, 2018 [26]	Systematic review	Psilocybin, LSD, and DPT	Patients with cancer-related psychiatric distress	10 studies	Cancer-related depression, anxiety, fear of death, psychological and existential distress, quality-of-life, and spiritual well-being
Alexander et al., 2025 [27]	Narrative review	Psilocybin and LSD	Patients with palliative care and with an advanced illness	Not reported	Existential distress, demoralization, quality of life, anxiety, depression, and suicide risk
Moshfeghinia et al., 2026 [28]	Systematic review and meta-analysis	Psilocybin	Patients with cancer	8 studies	Anxiety, depression, and other mental outcomes
Ferreira et al., 2025 [29]	Systematic review	Psilocybin, MDMA, LSD, mescaline, DPT and	Patients with life-threatening, incurable, or terminal illnesses and “palliative care	6 studies	Spirituality, spiritual well-being and mystical-type experience
Bader et al., 2024 [30]	Systematic review and meta-analysis	Psilocybin	Patients with advanced cancer	7 studies	Quality of life, pain control, and anxiety relief
Ko et al., 2022 [31]	Systematic Review	Psilocybin, ayahuasca, or ketamine	Patients with psychiatric and/or addictive disorders	12 studies	Association between mystical-type experience and symptom reduction
Kratina et al., 2026 [32]	Scoping review	Ketamine, psilocybin, ayahuasca, MDMA, DPT, LSD, ibogaine and mescaline	Patients with psychological suffering associated with life-threatening illness	59 studies	Outcome measures spanned biopsychosocial-spiritual domains
Amaev et al., 2025 [33]	Systematic review and meta-analysis	Serotonergic psychedelic-assisted therapy, psilocybin, LSD and MDMA	Patients with life-threatening illness	5 studies	Death anxiety, illness anxiety and State Trait Anxiety Inventory
Thivya Turner and Paul Glue 2025 [34]	Systematic Review	Psilocybin, MDMA, LSD, and DPT	Patients with life-threatening illness	14 studies	Outcome measures related to attitudes toward life, death, and spirituality
Sholevar et al., 2025 [35]	Narrative Review	Ketamine and ketamine-assisted psychotherapy (KAP)	Patients with serious medical illness	9 studies and 12 case reports	Psychiatric and existential distress

DMT = N,N-dimethyltryptamine; DPT = N,N-dipropyltryptamine; KAP = ketamine-assisted psychotherapy; LSD = lysergic acid diethylamide; MDMA = 3,4-methylenedioxymethamphetamine; HIV = human immunodeficiency virus.

**Table 2:** Overview of framework domains addressed in the included reviews of psychedelic-assisted interventions in palliative care.

Author, Year	Length of Therapy	Important Indications	Intrinsic Motivation	Mystical-Type Experiences	Integration with PC Healthcare Model	Personal or Family Issues	Clinical Training	Biographical and Relational Questions	Dosing and Administration
Maia et al., 2022 [11]	Explicit	Explicit	Partial	Explicit	Absent	Explicit	Explicit	Explicit	Explicit
Belitzky et al., 2025 [16]	Partial	Explicit	Absent	Absent	Partial	Absent	Partial	Absent	Explicit
Reiche et al., 2018 [17]	Absent	Explicit	Absent	Explicit	Partial	Partial	Absent	Explicit	Explicit
Schuman, et al., 2025 [18]	Absent	Explicit	Absent	Explicit	Partial	Absent	Partial	Partial	Explicit
Marchi, et al., 2024 [19]	Explicit	Explicit	Absent	Explicit	Partial	Absent	Absent	Partial	Explicit
Barbosa et al., 2023 [20]	Partial	Explicit	Absent	Absent	Explicit	Absent	Absent	Absent	Explicit
Jing, Hoeh & Menkes, 2022 [9]	Absent	Explicit	Absent	Explicit	Explicit	Absent	Absent	Absent	Explicit
Schimmers et al., 2022 [21]	Absent	Explicit	Absent	Explicit	Explicit	Explicit	Absent	Explicit	Explicit
Yu et al., 2021 [22]	Explicit	Explicit	Absent	Partial	Explicit	Absent	Absent	Absent	Explicit
Schipper et al., 2024 [23]	Explicit	Explicit	Absent	Absent	Explicit	Absent	Explicit	Absent	Explicit
White et al., 2023 [24]	Partial	Explicit	Absent	Explicit	Partial	Partial	Explicit	Partial	Explicit
Lapid et al., 2025 [25]	Partial	Explicit	Absent	Explicit	Partial	Partial	Explicit	Explicit	Explicit
Stephen Ross, 2018 [26]	Absent	Explicit	Absent	Explicit	Partial	Absent	Partial	Explicit	Explicit
Alexander et al., 2025 [27]	Explicit	Explicit	Absent	Explicit	Partial	Absent	Absent	Partial	Explicit
Moshfeghinia et al., 2026 [28]	Partial	Explicit	Absent	Absent	Partial	Absent	Partial	Partial	Explicit
Ferreira et al., 2025 [29]	Explicit	Explicit	Explicit	Explicit	Explicit	Explicit	Partial	Explicit	Explicit
Bader et al., 2024 [30]	Absent	Explicit	Absent	Partial	Partial	Absent	Absent	Partial	Explicit
Ko et al., 2022 [31]	Absent	Explicit	Absent	Explicit	Absent	Absent	Absent	Partial	Explicit
Kratina et al., 2026 [32]	Explicit	Explicit	Partial	Explicit	Explicit	Partial	Partial	Explicit	Explicit
Amaev et al., 2025 [33]	Explicit	Explicit	Partial	Absent	Absent	Absent	Absent	Absent	Explicit
Thivya Turner and Paul Glue 2025 [34]	Explicit	Explicit	Explicit	Explicit	Partial	Explicit	Partial	Explicit	Explicit
Sholevar et al., 2025 [35]	Explicit	Explicit	Partial	Explicit	Partial	Partial	Partial	Partial	Explicit

## Data Availability

No new data were created or analyzed in this study. Data sharing is not applicable to this article.

## Supplementary Materials

The following supporting information can be downloaded at: https://www.mdpi.com/article/10.3390/healthcare14111550/s1, Table S1: Search strategies in databases for psychedelics; Table S2: Excluded studies; Table S3: Proposed operational definitions for framework domains; Table S4: Extended characteristics of included studies, including findings, safety, and methodological considerations [9,11,16,17,18,19,20,21,22,23,24,25,26,27,28,29,30,31,32,33,34,35]; Table S5: Overview of framework domains addressed in the included reviews of psychedelic-assisted interventions in palliative care [9,11,16,17,18,19,20,21,22,23,24,25,26,27,28,29,30,31,32,33,34,35].

## References

[1] Saunders C. (1963). Management of intractable pain. Proc. R. Soc. Med..

[2] Bernstein I. (2022). At the intersection of palliative care, psychedelic medicine, and healthcare reform: A call for a new hospice and palliative care movement. J. Palliat. Care.

[3] World Health Organization WHO Definition of Palliative Care. https://www.who.int/cancer/palliative/definition/en/.

[4] Rosenbaum D., Boyle A.B., Rosenblum A.M., Ziai S., Chasen M.R. (2019). Psychedelics for psychological and existential distress in palliative and cancer care. Curr. Oncol..

[5] Kim A., Halton B., Shah A., Seecof O.M., Ross S. (2024). Psilocybin-Assisted Psychotherapy for Existential Distress: Practical Considerations for Therapeutic Application—A Review. Ann. Palliat. Med..

[6] Rosa W.E., Sager Z., Miller M., Bernstein I., Rinaldi A.D., Addicott K., Ljuslin M., Adrian C., Back A.L., Beachy J. (2022). Top Ten Tips Palliative Care Clinicians Should Know About Psychedelic-Assisted Therapy in the Context of Serious Illness. J. Palliat. Med..

[7] Sanders J.J., Beaussant Y. (2025). Feeling Groovy? The Present and Future of Psychedelic Research in Palliative Care. Palliat. Med..

[8] Li H., Wang X. (2025). Exploring End-of-Life Experiences and Consciousness through the Lens of Psychedelics. ACS Pharmacol. Transl. Sci..

[9] Jing X., Hoeh N.R., Menkes D.B. (2023). Psychedelic Medicines for End-of-Life Care: Pipeline Clinical Trial Review 2022. Palliat. Support. Care.

[10] Schuldt A., Clark I.C., Schmid Y., Ljuslin M., Boehlke C., Schipper S., Sands M.B., Blum D. (2025). Psychedelic-Assisted Therapy in Palliative Care—Insights from an International Workshop. Healthcare.

[11] Maia L.O., Beaussant Y., Garcia A.C.M. (2022). The therapeutic potential of psychedelic-assisted therapies for symptom control in patients diagnosed with serious illness: A systematic review. J. Pain Symptom Manag..

[12] Rosenbaum D., Hales S., Buchman D.Z. (2023). Commentary: Access to Psychedelics for Psychological Suffering at the End of Life—Prioritizing Our Priorities. Healthc. Policy.

[13] Beaussant Y., Tulsky J.A., Guérin B., Schwarz-Plaschg C., Sanders J.J. (2021). Mapping an Agenda for Psychedelic-Assisted Therapy Research in Patients with Serious Illness. J. Palliat. Med..

[14] Dyck E. (2019). Psychedelics and dying care: A historical look at the relationship between psychedelics and palliative care. J. Psychoact. Drugs.

[15] Page M.J., McKenzie J.E., Bossuyt P.M., Boutron I., Hoffmann T.C., Mulrow C.D., Shamseer L., Tetzlaff J.M., Akl E.A., Brennan S.E. (2021). The PRISMA 2020 statement: An updated guideline for reporting systematic reviews. BMJ.

[16] Belitzky E., Carvalho L.V.R., Taylor M., Ortiz C.N., Baum L., Fiellin D.A., Lustberg M.B. (2025). Psychedelics for cancer pain and associated psychological distress: A narrative review of a potential strategy. Cancer Med..

[17] Reiche S., Hermle L., Gutwinski S., Jungaberle H., Gasser P., Majić T. (2018). Serotonergic hallucinogens in the treatment of anxiety and depression in patients suffering from a life-threatening disease: A systematic review. Prog. Neuropsychopharmacol. Biol. Psychiatry.

[18] Schuman H.D.M., Savard C., Mina R., Barkova S., Conradi H.S.W., Deleemans J.M., Carlson L.E. (2025). Psychedelic-assisted therapies for psychosocial symptoms in cancer: A systematic review and meta-analysis. Curr. Oncol..

[19] Marchi M., Farina R., Rachedi K., Laonigro F., Žuljević M.F., Pingani L., Ferrari S., Somers M., Boks M.P.M., Galeazzi G.M. (2024). Psychedelics as an intervention for psychological and existential distress in terminally ill patients: A systematic review and network meta-analysis. J. Psychopharmacol..

[20] Barbosa M.G., Garcia G.T., Sarin L.M., Jackowski A.P. (2023). Efficacy and safety of ketamine for the treatment of depressive symptoms in palliative care: A systematic review. Braz. J. Psychiatry.

[21] Schimmers N., Breeksema J.J., Smith-Apeldoorn S.Y., Veraart J., van den Brink W., Schoevers R.A. (2022). Psychedelics for the treatment of depression, anxiety, and existential distress in patients with a terminal illness: A systematic review. Psychopharmacology.

[22] Yu C.-L., Yang F.-C., Yang S.-N., Tseng P.-T., Stubbs B., Yeh T.-C., Hsu C.-W., Li D.-J., Liang C.-S. (2021). Psilocybin for end-of-life anxiety symptoms: A systematic review and meta-analysis. Psychiatry Investig..

[23] Schipper S., Nigam K., Schmid Y., Piechotta V., Ljuslin M., Beaussant Y., Schwarzer G., Boehlke C. (2024). Psychedelic-assisted therapy for treating anxiety, depression, and existential distress in people with life-threatening diseases. Cochrane Database Syst. Rev..

[24] White C.M., Weisman N., Dalo J. (2023). Psychedelics for patients with cancer: A comprehensive literature review. Ann. Pharmacother..

[25] Lapid M.I., Pagali S.R., Randall A.L., Donovan K.A., Bronars C.A., Gauthier T.A., Bock J., Lim S.D., Carey E.C., Sokolowski E. (2025). Evaluating the effectiveness of psilocybin in alleviating distress among cancer patients: A systematic review. Palliat. Support. Care.

[26] Ross S. (2018). Therapeutic use of classic psychedelics to treat cancer-related psychiatric distress. Int. Rev. Psychiatry.

[27] Alexander W.B., Hansen E.D., Anderson B.T., Zarrabi A.J., Rogers A.H., Loewen G., Ficarro Z.R., Alexander M.H., Schaefer D., Case A.A. (2026). Meaning and psychedelics in palliative care: A narrative review. J. Pain Symptom Manag..

[28] Moshfeghinia R., Mostafavi S., Jazi K., Ghasemi A.R., Khosravaninezhad Y., Narayanan S., Ahmadi J., Pasalar M. (2026). The effects of psilocybin on psychological distress in cancer patients: A systematic review and meta-analysis. BMC Psychol..

[29] Ferreira A.E., Reis Pina P. (2025). Exploring the role of psychedelic assisted therapy in enhancing spirituality and mystical experiences in patients with life-threatening illnesses: A systematic review. J. Psychosom. Res..

[30] Bader H., Farraj H., Maghnam J., Abu Omar Y. (2024). Investigating the therapeutic efficacy of psilocybin in advanced cancer patients: A comprehensive review and meta-analysis. World J. Clin. Oncol..

[31] Ko K., Knight G., Rucker J.J., Cleare A.J. (2022). Psychedelics, mystical experience, and therapeutic efficacy: A systematic review. Front. Psychiatry.

[32] Kratina S., Strike C., Schwartz R., Nayfeh A., Jopling S., Lo C., Rush B. (2026). A scoping review of variations in psychedelic interventions for psychological suffering associated with the end of life. Soc. Sci. Med..

[33] Amaev A., Song J., Kambari Y., Carmona Torres E., Abdolizadeh A., Ueno F., Koizumi T., Strafella A.P., Husain M.I., Graff-Guerrero A. (2025). The effects of psychedelic-assisted therapy on illness and death anxiety: A systematic review and meta-analysis. J. Psychiatr. Res..

[34] Turner T., Glue P. (2025). Systematic review of the effect of psychedelic-assisted therapy on attitudes toward death, life, and spirituality on symptoms of distress, depression, and/or anxiety in patients with life-threatening illness. Psychedelic Med..

[35] Sholevar R., Kromka W., Beaussant Y. (2025). Ketamine and ketamine-assisted psychotherapy for psychiatric and existential distress in patients with serious medical illness: A narrative review. J. Palliat. Med..

[36] Knudsen G.M. (2022). Sustained Effects of Single Doses of Classical Psychedelics in Humans. Neuropsychopharmacology.

[37] McCulloch D.E.-W., Grzywacz M.Z., Madsen M.B., Jensen P.S., Ozenne B., Armand S., Knudsen G.M., Fisher P.M., Stenbæk D.S. (2022). Psilocybin-Induced Mystical-Type Experiences Are Related to Persisting Positive Effects: A Quantitative and Qualitative Report. Front. Pharmacol..

[38] Roméo B., Kervadec E., Fauvel B., Strika-Bruneau L., Amirouche A., Aurore B., Piolino P., Benyamina A. (2025). The Intensity of the Psychedelic Experience Is Reliably Associated with Clinical Improvements: A Systematic Review and Meta-Analysis. Neurosci. Biobehav. Rev..

[39] Brudner R.M., Kaczmarek E., Blainey M.G., Schulz C., Meshkat S., Doyle Z., Lipsitz O., Offman H., Sethi R., Weiglein G. (2025). Examining Mystical Experiences as a Predictor of Psilocybin-Assisted Psychotherapy for Treatment-Resistant Depression. J. Psychopharmacol..

[40] Roseman L., Nutt D., Carhart-Harris R. (2018). Quality of Acute Psychedelic Experience Predicts Therapeutic Efficacy of Psilocybin for Treatment-Resistant Depression. Front. Pharmacol..

[41] Weiss B., Roseman L., Giribaldi B., Nutt D., Carhart-Harris R., Erritzøe D. (2024). Unique Psychological Mechanisms Underlying Psilocybin Therapy Versus Escitalopram Treatment in the Treatment of Major Depressive Disorder. Int. J. Ment. Health Addict..

[42] Corman M., Dambrun M., Ginzac A., Ménard K. (2025). Exploring the Concept of Total Pain in Contemporary Oncology Palliative Care: A Qualitative Study on Patients’ Resources. BMC Palliat. Care.

[43] Malone T.C., Mennenga S.E., Guss J., Podrebarac S.K., Owens L.T., Bossis A.P., Belser A.B., Agin-Liebes G., Bogenschutz M.P., Ross S. (2018). Individual Experiences in Four Cancer Patients Following Psilocybin-Assisted Psychotherapy. Front. Pharmacol..

[44] Reynolds L., Barnett B.S., Weleff J., Morunga E., Wells A., Stack A., Akroyd A., Hoeh N., Sundram F., Muthukumaraswamy S. (2022). The Perceptions of Cancer Health-Care Practitioners in New Zealand and the USA toward Psychedelic-Assisted Therapy with Cancer Patients: A Cross-Sectional Survey. Palliat. Support. Care.

[45] Dorval M., Chang S.-L., Farzin H., Nguyen O., Stephan J.-F., Tapp D., Deschamps P., Joly Y., Moureaux F., Foxman R. (2025). Roadmap for Equitable Access and Responsible Use of Psilocybin-Assisted Psychotherapy in Palliative Care. Palliat. Med. Rep..

[46] Muthukumaraswamy S., Forsyth A., Lumley T. (2021). Blinding and Expectancy Confounds in Psychedelic Randomized Controlled Trials. Expert Rev. Clin. Pharmacol..

[47] Carhart-Harris R., Roseman L., Haijen E., Erritzøe D., Watts R., Branchi I., Kaelen M. (2018). Psychedelics and the Essential Importance of Context. J. Psychopharmacol..

[48] Niles H., Fogg C., Kelmendi B., Lazenby M. (2021). Palliative Care Provider Attitudes toward Existential Distress and Treatment with Psychedelic-Assisted Therapies. BMC Palliat. Care.

[49] Johnston C.B., Mangini M., Grob C.S., Anderson B. (2022). The Safety and Efficacy of Psychedelic-Assisted Therapies for Older Adults: Knowns and Unknowns. Am. J. Geriatr. Psychiatry.

[50] Bouchet L., Sager Z., Yrondi A., Nigam K., Anderson B., Ross S., Petridis P., Beaussant Y. (2024). Older Adults in Psychedelic-Assisted Therapy Trials: A Systematic Review. J. Psychopharmacol..

[51] Penn A., Phelps J., Rosa W.E., Watson J. (2021). Psychedelic-Assisted Psychotherapy Practices and Human Caring Science: Toward a Care-Informed Model of Treatment. J. Humanist. Psychol..

[52] Sebastiani E., Scacchetti M., Cesare M., Maurici M., Loiudice M.T. (2024). Identifying the Bundle/Care Development Process in Clinical Risk Management: A Systematic Review. Healthcare.

[53] Damschroder L.J., Reardon C.M., Opra Widerquist M.A., Lowery J. (2022). Conceptualizing outcomes for use with the Consolidated Framework for Implementation Research (CFIR): The CFIR Outcomes Addendum. Implement Sci..

