# Supplementary material for: Psychedelic-Assisted Interventions in Palliative Care: A Narrative Overview and Critical Evaluation
Source: Healthcare (Basel). 2026 Jun 2;14(11):1550. doi: 10.3390/healthcare14111550 (PMC13256648; doi:10.3390/healthcare14111550)
Supplement: Supplementary file 1 [file healthcare-14-01550-s001.zip › healthcare-4296027-supplementary.pdf]

healthcare MDPI

Table S1. Search strategies in databases for psychedelics

Search date: 02/02/2026

Psilocybin

MEDLINE via Pubmed

("Palliative Care"[Mesh] OR "Hospice Care"[Mesh] OR "palliative care" OR "end-of-life care" OR "terminal care" OR "Neoplasms"[Mesh] OR cancer OR oncology)

AND

("psilocybin"[All Fields] OR "psilocybin"[MeSH Terms] OR Psilocybin[Text Word] OR Psilocybine OR Psilocibin)

Results: 228

EMBASE via Ovid

1 Palliative Care.mp. or palliative therapy/ 151423

2 hospice care/ or Hospice Care.mp. 17842

3 terminal care/ or end-of-life care.mp. 55368

4 Neoplasms.mp. or neoplasm/ 770145

5 cancer.mp. or malignant neoplasm/ 5271577

6 oncology.mp. or oncology/ 448992

7 1 or 2 or 3 or 4 or 5 or 6 5720481

8 psilocybin.mp. or psilocybine/ 4620

9 7 and 8 365

Results: 365

 https://doi.org/10.3390/healthcare14111550

**LILACS**
("Palliative Care" OR “Cuidados Paliativos” OR "Hospice Care" OR "palliative care" OR "end-of-life care" OR "terminal care" OR "Neoplasms" OR “cancer” OR “oncology” OR “Neoplasias” OR “Câncer” OR “oncologia” OR "Cáncer" OR "Oncología")<br>AND<br>("psilocybin" OR “psilocibina” OR "psilocybin" OR “Psilocybin” OR “Psilocybine” OR “Psilocibin”)<br>**Results: 4**
**SCOPUS**
("palliative care" OR "hospice care" OR "end-of-life care" OR "terminal care" OR neoplasms OR cancer OR oncology) AND (psilocybin OR psilocybine OR psilocibin)<br>**Results: 314**
**Web of Science**
("palliative care" OR "hospice care" OR "end-of-life care" OR "terminal care" OR cancer OR oncology OR neoplasm*) AND (psilocybin OR psilocybine OR psilocibin)<br>**Results: 575**
**Ketamine (racemic)**
**MEDLINE via Pubmed**
("Palliative Care"[Mesh] OR "Hospice Care"[Mesh] OR "palliative care" OR "end-of-life care" OR "terminal care" OR "Neoplasms"[Mesh] OR cancer OR oncology)<br>AND<br>("ketamine"[All Fields] OR "esketamine"[All Fields] OR "Esketamine"[All Fields] OR "ketamine"[MeSH Terms] OR Ketamine[Text Word] OR “2-(2-Chlorophenyl)-2-(methylamino)cyclohexanone” OR Ketalar OR “CI-581” OR “CI581” OR “CI 581” OR Ketanest OR “Ketamine Hydrochloride” OR Calipsol OR Calypsol OR Kalipsol OR Ketaset)<br>**Results: 1452**
**EMBASE via Ovid**
1 Palliative Care.mp. or palliative therapy/ 151423<br>2 hospice care/ or Hospice Care.mp. 17842

3 terminal care/ or end-of-life care.mp. 55368
4 Neoplasms.mp. or neoplasm/ 770145
5 cancer.mp. or malignant neoplasm/ 5271577
6 oncology.mp. or oncology/ 448992
7 1 or 2 or 3 or 4 or 5 or 6 5720481
8 ketamine.mp. or ketamine/ 83905
9 7 and 8 4940
**Results: 4940**
**LILACS**
("Palliative Care" OR “Cuidados Paliativos” OR "Hospice Care" OR "palliative care" OR "end-of-life care" OR "terminal care" OR "Neoplasms" OR “cancer” OR “oncology” OR “Neoplasias” OR “Câncer” OR “oncologia” OR "Cáncer" OR "Oncología") AND ("ketamine" OR “cetamina” OR “ketamina” OR "esketamine" OR "Esketamine" OR "ketamine" OR “Ketamine” OR “2-(2-Chlorophenyl)-2-(methylamino)cyclohexanone” OR “Ketalar” OR “CI-581” OR “CI581” OR “CI 581” OR “Ketanest” OR “Ketamine Hydrochloride” OR “Calipsol” OR “Calypsol” OR “Kalipsol” OR “Ketaset”)
**Results: 56**
**SCOPUS**
("palliative care" OR "hospice care" OR "end-of-life care" OR "terminal care" OR neoplasms OR cancer OR oncology) AND (ketamine OR esketamine OR "2-(2-chlorophenyl)-2-(methylamino)cyclohexanone" OR ketalar OR "CI-581" OR "CI581" OR "CI 581" OR ketanest OR "ketamine hydrochloride" OR calipsol OR calypsol OR kalipsol OR ketaset)
**Results: 4954**
**Web of Science**
("palliative care" OR "hospice care" OR "end-of-life care" OR "terminal care" OR cancer OR oncology OR neoplasm*) AND

(ketamine OR esketamine OR "2-(2-chlorophenyl)-2-(methylamino)cyclohexanone" OR ketalar OR ketanest OR "CI-581" OR "CI581" OR "CI 581" OR "ketamine hydrochloride" OR calipsol OR calypsol OR kalipsol OR ketaset)

Results: 295

LSD (lysergic acid diethylamide)

MEDLINE via Pubmed

("Palliative Care"[Mesh] OR "Hospice Care"[Mesh] OR "palliative care" OR "end-of-life care" OR "terminal care" OR "Neoplasms"[Mesh] OR cancer OR oncology)

AND

("lysergic acid diethylamide"[All Fields] OR "lysergic acid diethylamide"[MeSH Terms] OR Lysergic Acid Diethylamide[Text Word] OR “LSD” OR “LSD-25” OR “LSD 25” OR Lysergide OR “Lysergic Acid Diethylamide Tartrate”)

Results: 455

EMBASE via Ovid

1Palliative Care.mp. or palliative therapy/ 151423

2 hospice care/ or Hospice Care.mp. 17842

3 terminal care/ or end-of-life care.mp. 55368

4 Neoplasms.mp. or neoplasm/ 770145

5 cancer.mp. or malignant neoplasm/ 5271577

6 oncology.mp. or oncology/ 448992

7 1 or 2 or 3 or 4 or 5 or 6 5720481

8 lysergide/ or lysergic acid diethylamide.mp. 9458

9 LSD.mp. or lysergide/ 15060

10 8 or 9 15199

11 7 and 10 653

Results: 653

**LILACS**
("Palliative Care" OR “Cuidados Paliativos” OR "Hospice Care" OR "palliative care" OR "end-of-life care" OR "terminal care" OR "Neoplasms" OR “cancer” OR “oncology” OR “Neoplasias” OR “Câncer” OR “oncologia” OR "Cáncer" OR "Oncología")<br>AND<br>("lysergic acid diethylamide" OR “dietilamida do ácido lisérgico” OR “dietilamida del ácido lisérgico” OR "lysergic acid diethylamide" OR “Lysergic Acid Diethylamide” OR “LSD” OR “LSD-25” OR “LSD 25” OR “Lysergide” OR “Lysergic Acid Diethylamide Tartrate”)<br>**Results: 14**
**SCOPUS**
("palliative care" OR "hospice care" OR "end-of-life care" OR "terminal care" OR neoplasms OR cancer OR oncology)<br>AND<br>("lysergic acid diethylamide" OR LSD OR "LSD-25" OR "LSD 25" OR lysergide OR "lysergic acid diethylamide tartrate")<br>**Results: 612**
**Web of Science**
("palliative care" OR "hospice care" OR "end-of-life care" OR "terminal care" OR cancer OR oncology OR neoplasm*) AND ("lysergic acid diethylamide" OR LSD OR "LSD-25" OR "LSD 25" OR lysergide OR "lysergic acid diethylamide tartrate")<br>**Results: 306**
**DMT (Dimethyltryptamine)/Ayahuasca**
**MEDLINE via Pubmed**
("Palliative Care"[Mesh] OR "Hospice Care"[Mesh] OR "palliative care" OR "end-of-life care" OR "terminal care" OR "Neoplasms"[Mesh] OR cancer OR oncology)<br>AND<br>("n, n-dimethyltryptamine"[All Fields] OR N, N-Dimethyltryptamine[Text Word] OR “N,N Dimethyltryptamine” OR Dimethyltryptamine)<br>**Results: 31**
**EMBASE via Ovid**

1 Palliative Care.mp. or palliative therapy/ 151423

2 hospice care/ or Hospice Care.mp. 17842

3 terminal care/ or end-of-life care.mp. 55368

4 Neoplasms.mp. or neoplasm/ 770145

5 cancer.mp. or malignant neoplasm/ 5271577

6 oncology.mp. or oncology/ 448992

7 1 or 2 or 3 or 4 or 5 or 6 5720481

8 dimethyltryptamine/ or n, n-dimethyltryptamine.mp. 2186

9 DMT.mp. 9145

10 8 or 9 10435

11 7 and 10 523

Results: 523

LILACS

("Palliative Care" OR “Cuidados Paliativos” OR "Hospice Care" OR "palliative care" OR "end-of-life care" OR "terminal care" OR "Neoplasms" OR “cancer” OR “oncology” OR “Neoplasias” OR “Câncer” OR “oncologia” OR "Cáncer" OR "Oncología")

AND

("n, n-dimethyltryptamine" OR “n, n-dimetiltriptamina” OR “N, N-Dimethyltryptamine” OR “N,N Dimethyltryptamine” OR “Dimethyltryptamine”)

Results: 6

SCOPUS

("palliative care" OR "hospice care" OR "end-of-life care" OR "terminal care" OR neoplasms OR cancer OR oncology)

AND

("n,n-dimethyltryptamine" OR "n,n dimethyltryptamine" OR dimethyltryptamine OR DMT)

Results: 315

**Web of Science**
("palliative care" OR "hospice care" OR "end-of-life care" OR "terminal care" OR cancer OR oncology OR neoplasm*) AND ("lysergic acid diethylamide" OR LSD OR "LSD-25" OR "LSD 25" OR lysergide OR "lysergic acid diethylamide tartrate")<br>**Results: 23**
**Mescaline**
**MEDLINE via Pubmed**
("Palliative Care"[Mesh] OR "Hospice Care"[Mesh] OR "palliative care" OR "end-of-life care" OR "terminal care" OR "Neoplasms"[Mesh] OR cancer OR oncology)<br>AND<br>("mescaline"[All Fields] OR "mescaline"[MeSH Terms] OR Mescaline[Text Word] OR "3,4,5-Trimethoxyphenethylamine" OR Trimethoxyphenethylamine OR Peyote OR Mezcalin)<br>**Results: 15**
**EMBASE via Ovid**
1 Palliative Care.mp. or palliative therapy/ 151423<br>2 hospice care/ or Hospice Care.mp. 17842<br>3 terminal care/ or end-of-life care.mp. 55368<br>4 Neoplasms.mp. or neoplasm/ 770145<br>5 cancer.mp. or malignant neoplasm/ 5271577<br>6 oncology.mp. or oncology/ 448992<br>7 1 or 2 or 3 or 4 or 5 or 6 5720481<br>8 mescaline/ or mescaline.mp. 2060<br>9 7 and 8 68<br>**Results: 523**
**LILACS**

("Palliative Care" OR “Cuidados Paliativos” OR "Hospice Care" OR "palliative care" OR "end-of-life care" OR "terminal care" OR "Neoplasms" OR “cancer” OR “oncology” OR “Neoplasias” OR “Câncer” OR “oncologia” OR "Cáncer" OR "Oncología")<br>AND<br>("mescaline" OR “mescalina” OR “3,4,5-Trimethoxyphenethylamine” OR “3,4,5-Trimetoxifenetilamina” OR “Trimethoxyphenethylamine” OR “Peyote” OR “Mezcalin”)<br>**Results: 1**
**SCOPUS**
("palliative care" OR "hospice care" OR "end-of-life care" OR "terminal care" OR neoplasms OR cancer OR oncology)<br>AND<br>(mescaline OR mezcalin OR peyote OR "3,4,5-trimethoxyphenethylamine" OR trimethoxyphenethylamine)<br>**Results: 64**
**Web of Science**
("palliative care" OR "hospice care" OR "end-of-life care" OR "terminal care" OR cancer OR oncology OR neoplasm*) AND (mescaline OR "3,4,5-trimethoxyphenethylamine" OR trimethoxyphenethylamine OR peyote OR mezcalin)<br>**Results: 8**
**MDMA**
**MEDLINE via Pubmed**
("Palliative Care"[Mesh] OR "Hospice Care"[Mesh] OR "palliative care" OR "end-of-life care" OR "terminal care" OR "Neoplasms"[Mesh] OR cancer OR oncology) AND (MDMA[Title/Abstract] OR "3,4-methylenedioxymethamphetamine"[All Fields])<br>**Results: 69**
**EMBASE via Ovid**
1 Palliative Care.mp. or palliative therapy/ 151423<br>2 hospice care/ or Hospice Care.mp. 17842

3 terminal care/ or end-of-life care.mp. 55368

4 Neoplasms.mp. or neoplasm/ 770145

5 cancer.mp. or malignant neoplasm/ 5271577

6 oncology.mp. or oncology/ 448992

7 1 or 2 or 3 or 4 or 5 or 6 5720481

8 midomafetamine/ or MDMA.mp. 12662

9 7 and 8 221

Results: 221

LILACS

("Palliative Care" OR “Cuidados Paliativos” OR "Hospice Care" OR "palliative care" OR "end-of-life care" OR "terminal care" OR "Neoplasms" OR “cancer” OR “oncology” OR “Neoplasias” OR “Câncer” OR “oncologia” OR "Cáncer" OR "Oncología")

AND

(“MDMA” OR "3,4-methylenedioxymethamphetamine" OR “3,4-metilenodioximetanfetamina” OR “3,4-metilendioximetanfetamina”)

Results: 1

SCOPUS

("palliative care" OR "hospice care" OR "end-of-life care" OR "terminal care" OR neoplasms OR cancer OR oncology)

AND

(MDMA OR "3,4-methylenedioxymethamphetamine")

Results: 197

Web of Science

("palliative care" OR "hospice care" OR "end-of-life care" OR "terminal care"OR cancer OR oncology OR neoplasm*) AND (MDMA OR "3,4-methylenedioxymethamphetamine")

Results: 79

**Ibogaine**
**MEDLINE via Pubmed**
("Palliative Care"[Mesh] OR "Hospice Care"[Mesh] OR "palliative care" OR "end-of-life care" OR "terminal care" OR "Neoplasms"[Mesh] OR cancer OR oncology) AND ("ibogaine"[All Fields] OR "ibogaine"[MeSH Terms] OR ibogaine[Text Word] OR "12-Methoxyibogamine" OR "12 Methoxyibogamine" OR Endabuse OR "NIH-10567" OR "NIH 10567")<br>**Results: 28**
**EMBASE via Ovid**
1 Palliative Care.mp. or palliative therapy/ 151423<br>2 hospice care/ or Hospice Care.mp. 17842<br>3 terminal care/ or end-of-life care.mp. 55368<br>4 Neoplasms.mp. or neoplasm/ 770145<br>5 cancer.mp. or malignant neoplasm/ 5271577<br>6 oncology.mp. or oncology/ 448992<br>7 1 or 2 or 3 or 4 or 5 or 6 5720481<br>8 ibogaine/ or ibogaine.mp 956<br>9 7 and 8 56<br>**Results: 56**
**LILACS**
("Palliative Care" OR "Cuidados Paliativos" OR "Hospice Care" OR "palliative care" OR "end-of-life care" OR "terminal care" OR "Neoplasms" OR "cancer" OR "oncology" OR "Neoplasias" OR "Câncer" OR "oncologia" OR "Cáncer" OR "Oncología")<br>AND<br>("ibogaine" OR "ibogaína" OR "12-Methoxyibogamine" OR "12 Methoxyibogamine" OR "Endabuse" OR "NIH-10567" OR "NIH 10567")<br>**Results: 1**

SCOPUS

("palliative care" OR "hospice care" OR "end-of-life care" OR "terminal care" OR neoplasms OR cancer OR oncology)

AND

(ibogaine OR endabuse OR "12-methoxyibogamine" OR "12 methoxyibogamine" OR "NIH-10567" OR "NIH 10567")

Results: 66

Web of Science

("palliative care" OR "hospice care" OR "end-of-life care" OR "terminal care" OR cancer OR oncology OR neoplasm*) AND (ibogaine OR "12-methoxyibogamine" OR "12 methoxyibogamine" OR endabuse OR "NIH-10567" OR "NIH 10567")

Results: 4

Esketamine

MEDLINE via Pubmed

("Palliative Care"[Mesh] OR "Hospice Care"[Mesh] OR "palliative care" OR "end-of-life care" OR "terminal care" OR "Neoplasms"[Mesh] OR cancer OR oncology) AND ("esketamine"[All Fields] OR esketamine[Text Word] OR "L-Ketamine" OR "(-)-Ketamine" OR "S-Ketamine" OR Kataved OR Spravato)

Results: 1445

EMBASE via Ovid

1 Palliative Care.mp. or palliative therapy/ 151423

2 hospice care/ or Hospice Care.mp. 17842

3 terminal care/ or end-of-life care.mp. 55368

4 Neoplasms.mp. or neoplasm/ 770145

5 cancer.mp. or malignant neoplasm/ 5271577

6 oncology.mp. or oncology/ 448992

7 1 or 2 or 3 or 4 or 5 or 6 5720481

8 esketamine.mp. or esketamine/ 3897

9 Spravato.mp 152
10 8 or 9 3900
11 7 and 10 225
**Results: 225**
**LILACS**
("Palliative Care" OR “Cuidados Paliativos” OR "Hospice Care" OR "palliative care" OR "end-of-life care" OR "terminal care" OR "Neoplasms" OR “cancer” OR “oncology” OR “Neoplasias” OR “Câncer” OR “oncologia” OR "Cáncer" OR "Oncología")
AND
("esketamine" OR “esketamina” OR “L-Ketamine” OR “(-)-Ketamine” OR “S-Ketamine” OR Kataved OR Spravato)
**Results: 96**
**SCOPUS**
("palliative care" OR "hospice care" OR "end-of-life care" OR "terminal care" OR neoplasms OR cancer OR oncology)
AND
(esketamine OR "l-ketamine" OR "(-)-ketamine" OR "s-ketamine" OR kataved OR spravato)
**Results: 4950**
**Web of Science**
("palliative care" OR "hospice care" OR "end-of-life care" OR "terminal care" OR cancer OR oncology OR neoplasm*) AND
(esketamine OR "L-ketamine" OR "(-)-ketamine" OR "S-ketamine" OR kataved OR spravato)
**Results: 295**

Table S2. Excluded studies.

Title	Author	Year	DOI	Reason for exclusion
**A protocol for a scoping review of variations among psychedelic interventions for psychological suffering associated with the end-of-life**	Kratina et al.	2025	10.1371/journal.pone.0318343	Type of publication (scoping review protocol)
**Use of ketamine in critically ill patients: a narrative review.**	Schuman et al.	2022	https://doi.org/10.5935/0103-507X.20220027-pt	Population outside of PICOT
**Dexmedetomidine in Palliative Care: A Versatile New Weapon Against Delirium and Pain-Systematic Review**	Tavares et al.	2024	10.1089/jpm.2023.0609	Intervention outside the scope
**Psychedelic-Assisted Therapy and Psychedelic Science: A Review and Perspective on Opportunities in Neurosurgery and Neuro-Oncology**	Kelly et al.	2023	10.1227/neu.0000000000002275	Type of publication (perspective/opinion)
**Update on treatments for anxiety-related disorders**	Lee et al.	2022	10.1097/YCO.0000000000000841	Type of publication
**Description of the use of ketamine for opioid-refractory pain in an inpatient palliative care population**	Callaghan et al.	2025	https://dx.doi.org/10.1093/ajhp/zxae345 PT - Conference Abstract	Type of publication (medical record review)
**Inhaled nebulised medications in palliative care - a survey among palliative care practitioners in Germany**	Mair et al.	2025	https://dx.doi.org/10.1186/s12904-025-01761-y PT - Article	Type of publication
**The diverse effects of ketamine, jack-of-all-trades: a narrative review**	Richards et al.	2025	https://dx.doi.org/10.1016/j.bja.2024.11.018	Population outside of PICOT
**Ketamine Use for Palliative Care in the Austere Environment: Is Ketamine the Path Forward for Palliative Care**	Reed et al.	2025	https://dx.doi.org/10.1177/10499091241246520 PT - Article	Type of publication
**Adjunctive Intranasal Esketamine for Comorbid Treatment-Resistant Depression with Suicidal Ideation in Patients Receiving Palliative Care at a Comprehensive Cancer Center: A Case Series**	Tomy et al.	2025	https://dx.doi.org/10.1089/jpm.2024.0040 PT - Article	Type of publication (case series)
**A literature review of management for opioid-refractory neuropathic cancer pain: an update and future perspectives**	Matsuoka et al.	2025	https://dx.doi.org/10.21037/amj-23-175 PT - Review	Intervention outside the scope
**Management of pain in cancer patients - an update**	Daud et al.	2024	https://dx.doi.org/10.3332/ecancer.2024.1821 PT - Article	Intervention outside the scope
**Ketamine for Super-Refractory Status Epilepticus in Palliative Care. A Case Report and Review of the Literature**	Dunn et al.	2024	https://dx.doi.org/10.1177/10499091231215491 PT - Article	Type of publication
**Scoping Review: The Role of Psychedelics in the Management of Chronic Pain**	Robinson et al.	2024	https://dx.doi.org/10.2147/JPR.S439348 PT - Review	Population outside of PICOT

**Intranasal Therapy in Palliative Care**	Ingielewicz et al.	2024	https://dx.doi.org/10.3390/pharmaceutics16040519 PT - Review	Intervention outside the scope
**Continuous palliative sedation until death: a qualitative study of palliative care clinicians' experiences**	Guite-Verret et al.	2024	https://dx.doi.org/10.1186/s12904-024-01426-2 PT - Article	Type of publication
**The American society of pain and neuroscience (Aspn) best practices and guidelines for the interventional management of cancer-associated pain**	Horowitz et al.	2024	https://dx.doi.org/10.1016/j.jpainsymman.2024.02.393 PT - Conference Abstract	Type of publication (conference abstract)
**Beyond the drugs-parental perspectives on managing multifactorial pain in paediatric palliative care**	Aman et al.	2021	https://dx.doi.org/10.2147/JPR.S315585 PT - Review	Intervention outside the scope
**Beyond the drugs-parental perspectives on managing multifactorial pain in paediatric palliative care**	Sprinz et al.	2021	https://dx.doi.org/10.1136/archdischild-2021-rcpch.140 PT - Conference Abstract	Type of publication
**The use of ketamine in the management of refractory cancer pain in a palliative care unit**	Cheung et al.	2020	https://dx.doi.org/10.21037/apm.2019.09.09 PT - Article	Type of publication
**Practice review: Evidence-based and effective management of pain in patients with advanced cancer**	Chapman et al.	2020	https://dx.doi.org/10.1177/0269216319896955 PT - Review	Intervention outside the scope
**Drug use beyond the licence in palliative care: A systematic review and narrative synthesis**	Hagemann et al.	2019	https://dx.doi.org/10.1177/0269216319840602 PT - Review	Intervention outside the scope
**A research study review of effectiveness of treatments for psychiatric conditions common to end-stage cancer patients: Needs assessment for future research and an impassioned plea**	Johnson et al.	2018	https://dx.doi.org/10.1186/s12888-018-1651-9 PT - Article	Type of publication
**Ketamine, an alternative in the anesthetic treatment of cancer patients**	Vazquez-Morales et al.	2018	https://dx.doi.org/10.24875/j.gamo.18000077 PT - Review	Intervention outside the scope
**Impact of a Comprehensive Ketamine Protocol on Cancer Pain in a Palliative Care Unit**	Meng et al.	2018	https://dx.doi.org/10.1016/j.jpainsymman.2018.10.182 PT - Conference Abstract	Type of publication (prospective cohort)
**Ketamine for the Treatment of Depression in Patients Receiving Hospice Care: A Retrospective Medical Record Review of Thirty-One Cases**	Iglewicz et al.	2015	https://dx.doi.org/10.1016/j.psym.2014.05.005 PT - Article	Type of publication
**Why do they call it special K? The how, when, why, and what-ifs of using ketamine in the palliative care setting**	Parikh et al.	2015		Type of publication
**Ketamine PCA for treatment of end-of-life neuropathic pain in pediatrics**	Taylor et al.	2015		Type of publication
**Pain Management at the End of Life in the Emergency Department: A Narrative Review of the Literature and a Practical Clinical Approach**	Serra et al.	2023	https://dx.doi.org/10.3390/jcm12134357 PT - Review	Intervention outside the scope

**MO28-5 Effectiveness of ketamine for depression among advanced cancer patients in palliative care: A systematic review**	Cipta et al.	2023	https://dx.doi.org/10.1016/j.annonc.2023.09.226 PT - Conference Abstract	Type of publication
**A systematic review of pharmacologic treatment efficacy for depression in older patients with cancer**	Rabin et al.	2022	https://dx.doi.org/10.1016/j.bbih.2022.100449 PT - Review	Intervention outside the scope
**Personalized Medicine for Classical Anesthesia Drugs and Cancer Progression**	Costa et al.	2022	https://dx.doi.org/10.3390/jpm12111846 PT - Review	Intervention outside the scope
**End-of-life care in children and adolescents with cancer: perspectives from a French pediatric oncology care network**	Blais et al.	2022	https://dx.doi.org/10.1177/03008916211013384 PT - Article	Type of publication
**Interventional Pain Management in Palliative Care**	Hawley et al.	2022	https://dx.doi.org/10.1007/978-3-030-86244-2_12 PT - Chapter	Type of publication
**Evidence-Based Management of Depression in Palliative Care: A Systematic Review**	Perusinghe et al.	2021	https://dx.doi.org/10.1089/jpm.2020.0659 PT - Review	Intervention outside the scope
**Update in hospice and palliative care**	Roza et al.	2014	https://dx.doi.org/10.1089/jpm.2013.0577 PT - Review	Intervention outside the scope
**Palliative care of people with oesophageal cancer**	Clark et al.	2011		Intervention outside the scope
**Mapping an Agenda for Psychedelic-Assisted Therapy Research in Patients with Serious Illness**	Beaussant et al.	2021	10.1089/jpm.2020.0764	Type of publication (qualitative analysis of expert opinion)
**Taking Psychedelics Seriously**	Ira Byock	2018	10.1089/jpm.2017.0684	Type of publication (Special report)
**Ayahuasca and cancer treatment**	Eduardo E Schenberg	2013	10.1177/2050312113508389	Type of publication (narrative/hypothetical article compiling nine case reports)
**Exploring End-of-Life Experiences and Consciousness through the Lens of Psychedelics**	Li & Wang	2025		Type of publication (opinion article / conceptual essay)
**Psychedelic research, assisted therapy and the role of the anaesthetist: A review and insights for experimental and clinical practices**	Lima et al.	2024	https://dx.doi.org/10.1111/bcp.16264 PT - Review	Population outside of PICOT
**Cancer in multiple sclerosis patients following prolonged exposure to disease-modifying therapies (DMTs): a systematic review and meta-analysis**	Giannopapas et al.	2025		Intervention outside the scope
**The Placebo Response in Classic Psychedelics: A Systematic Review of Clinical Trials and Qualitative Analysis**	Weissman et al.	2022		Type of publication
**Psychedelics: A new era of treatment?**	Torres S.	2021	https://dx.doi.org/10.1192/j.eurpsy.2021.1290 PT - Conference Abstract	Type of publication
**Psychedelics for psychological and existential distress in palliative and cancer care**	Rosenbaum et al.	2019	10.3747/co.26.5009	Type of publication

**Opening doors of perception: Psychedelic drugs and end-of-life care**	MacReady N.	2012	https://dx.doi.org/10.1093/jnci/djs468 PT - Note	Type of publication
**Ketamina en el manejo del dolor oncológico**	Pardo et al.	2022		Population outside of PICOT
**End of Life with Psylocibin: Research, Data and Experience**	T. Re, A. Metastasio	2025	https://doi.org/10.1016/j.etdah.2025.100250	Type of publication (conference poster)
**Low-Dose Psilocybin Therapy for Palliative Care Patients With Chronic Cancer Pain Requiring Opioids**		2025	https://clinicaltrials.gov/study/NCT06827054 PT - Clinical Trial	Type of publication
**Psychedelic-Assisted Therapies in Psychosocial Oncology: Opportunities and Challenges**	Sholevar R.	2024	https://dx.doi.org/10.1002/pon.6287 PT - Conference Abstract	Type of publication (conference poster)
**A Review of Psychedelic-Assisted Psychotherapy for Cancer Patients with Emotional Distress**	Tourgeman et al.	2024	https://dx.doi.org/10.1016/j.apmr.2024.02.560 PT - Conference Abstract	Type of publication (conference poster)
**TRIP - TReatment to Improve Depression and/or Anxiety Using Psilocybin-Assisted Psychotherapy in Patients With Advanced Cancer on Maintenance Therapy**		2023	https://clinicaltrials.gov/study/NCT06200155 PT - Clinical Trial	Type of publication (clinical study)
**Psilocybin-assisted therapy mediates psycho-social-spiritual change in cancer patients as assessed by the NIH-HEALS**	Shnayder et al.	2023		Type of publication (clinical study)
**Systematized Review of Psychotherapeutic Components of Psilocybin-Assisted Psychotherapy**	Horton et al.	2021	10.1176/appi.psychotherapy.20200055	Type of publication (clinical study)
**Psilocybin-assisted group therapy in patients with cancer diagnosed with a major depressive disorder.**	Agrawal et al.	2024	10.1002/cncr.35010	Population outside of PICOT
**Innovations in group-based psilocybin-assisted therapy of major depression in patients with cancer.**	Thrul et al.	2024		Type of publication
**Psychedelic-Assisted Therapy and Psychedelic Science: A Review and Perspective on Opportunities in Neurosurgery and Neuro-Oncology**	Kelly et al.	20232	https://doi.org/10.1227/neu.0000000000002275	Population outside of PICOT
**The Placebo Response in Classic Psychedelics: A Systematic Review of Clinical Trials and Qualitative Analysis**	Weissman et al.	2022	https://dx.doi.org/10.1038/s41386-022-01486-z PT - Conference Abstract	Type of publication (conference poster)
**Palliative care: Is it time for health professionals to talk openly about psychedelic therapy?**	Presern E.	2021	https://dx.doi.org/10.3399/bjgp21X716333 PT - Note	Type of publication (editorial)
**Psychedelic and related medicines at the end of life**		20212	https://journal.nzma.org.nz/journal-articles/psychedelic-and-related-medicines-at-the-end-of-life PT - Letter	Type of publication (letter to the editor)

**The top ten things that must be known about end of life therapy in patients with advanced cancer**	Kreye et al.	2020		Type of publication
**Psychedelica bij existentieel lijden bij patiënten met een terminale ziekte**	SCHIMMEL et al.	2020		Language (Dutch)
**Psychedelics and Dying Care: A Historical Look at the Relationship between Psychedelics and Palliative Care**	Dyck E.	2019	https://dx.doi.org/10.1080/02791072.2019.1581308 PT - Article	Type of publication
**A novel group psychotherapy modality for psychosocial distress that employs the psychotherapeutic catalyst psilocybin**	Anderson et al.	2016	https://dx.doi.org/10.1002/pon.4082 PT - Conference Abstract	Type of publication
**Exploring the Use of Psilocybin Therapy for Existential Distress: A Qualitative Study of Palliative Care Provider Perceptions**	Mayer et al.	2022	10.1080/02791072.2021.1916659	Type of publication
**Examining the Rationale for Studying Psychedelic-Assisted Psychotherapy for the Treatment of Caregiver Distress**	Gold et al.	2023	10.1089/psymed.2022.0011	Population outside of PICOT
**Antidepressant effect of intranasal ketamine in the palliative care of terminally ill cancer patients**	González-Pardo et al.	2023	10.3390/cancers15020400	Type of publication
**Unfolding States of Mind: A Dissociative-Psychedelic Model of Ketamine-Assisted Psychotherapy in Palliative Care**	Campolina et al.	2025	10.3390/healthcare13212714	Type of publication
**Contributions of the compassionate care approach to psychedelic-assisted therapiesin hospice and palliative care**	Garcia et al.	2024	https://doi.org/10.1080/09699260.2024.2374602	Type of publication
**Psilocybin in Palliative Care: An Update**	Whinkin et al.	2023	10.1007/s13670-023-00383-7	Type of publication
**Psychedelic-assisted psychotherapy to treat psychiatric and existential distress in life-threatening medical illnesses and palliative care**	Ross et al.	2022	https://doi.org/10.1016/j.neuropharm.2022.109174	Type of publication
**Psilocybin-assisted psychotherapy for existential distress: practical considerations for therapeutic application—a review**	Kim et al.	2024	doi: 10.21037/apm-24-35	Type of publication
**The Potential of Psychedelics for End of Life and Palliative Care**	Yaden et al.	2022	doi: 10.1007/7854_2021_278.	Type of publication
**Psychedelics for the treatment of end-of-life distress in patients with a life-threatening disease**	Tap et al.	2025	10.1016/bs.irn.2025.03.001	Type of publication
**Psychedelic-assisted therapies for existential and spiritual suffering in palliative care**	Ana Cláudia Mesquita Garcia, Lucas Oliveira Maia	2025	10.1016/bs.pbr.2025.07.002	Type of publication

Table S3 – Proposed operational definitions for framework domains

Framework Domain	Operational Definition	Operational Focus	Examples of Explicit Coverage
**1-Length of Therapy**	Extent to which the review discusses the temporal structure and continuity of psychedelic-assisted therapy (PAT), including the number and sequencing of preparatory, dosing, and integration sessions, duration of follow-up, and longitudinal organization of care.	Preparation phase duration; number and frequency of dosing sessions; integration sessions; follow-up care; continuity and pacing of treatment.	Discussion of multi-session models, staged preparation-integration processes, or longitudinal therapeutic trajectories.
**2-Important Indications**	Extent to which the review identifies or discusses clinical indications, target populations, or symptom clusters considered appropriate or potentially beneficial for PAT within palliative care contexts.	Existential distress; depression; anxiety related to life-threatening illness; demoralization; spiritual distress; end-of-life suffering; cancer-related psychological distress.	Discussion of diagnostic criteria, patient selection rationale, or clinical contexts where PAT may be indicated.
**3-Intrinsic Motivation**	Extent to which the review addresses the patient's internal willingness, psychological readiness, openness, intentionality, or active engagement in the therapeutic process.	Treatment readiness; psychological openness; intentional engagement; voluntary participation; meaning-making orientation; motivation for psychological or existential exploration.	References to patient preparedness, intentional therapeutic engagement, or motivational factors influencing outcomes.
**4-Mystical-type Experiences**	Extent to which the review discusses subjective altered states commonly described as mystical, transcendent, sacred, spiritually significant, or characterized by unity, ego dissolution, interconnectedness, or profound meaning.	Mystical experience; oceanic boundlessness; ego dissolution; spiritual significance; transcendence; sacredness; existential insight.	Discussion of mystical experience questionnaires, associations between mystical experiences and outcomes, or therapeutic significance of transcendent states.
**5-Integration with Palliative Care Healthcare Model**	Extent to which the review discusses how PAT may be incorporated into existing palliative care structures, workflows, interdisciplinary teams, and continuity-of-care models.	Interdisciplinary collaboration; continuity of care; integration with oncology or hospice care; referral pathways; coordination with palliative care services; spiritual and psychosocial support systems.	Discussion of institutional implementation, integration into palliative care settings, or coordination between PAT providers and palliative care teams.
**6-Personal or Family Issues**	Extent to which the review addresses the role of interpersonal, familial, social, or unresolved personal issues within the therapeutic process or clinical outcomes.	Family dynamics; relational suffering; grief; unresolved conflicts; attachment-related themes; caregiver relationships; interpersonal reconciliation.	Discussion of family involvement, relational healing, interpersonal distress, or psychosocial dimensions of end-of-life care.
**7-Clinical Training**	Extent to which the review discusses professional competencies, therapist preparation, supervision, ethical training, or institutional requirements necessary for safe and effective delivery of PAT.	Therapist competencies; specialized training; ethical preparation; supervision; interdisciplinary competencies; safety procedures; credentialing.	Discussion of therapist training standards, competency-based education, or professional qualification requirements.
**8-Biographical and Relational Questions**	Extent to which the review addresses the importance of exploring the patient's life history, relational experiences, identity, personal meaning, or existential narrative within the therapeutic process.	Personal history; trauma history; existential biography; relational patterns; identity and meaning; narrative exploration; life review processes.	Discussion of biographical interviewing, life-story exploration, relational context, or existential narrative work during preparation or integration.
**9-Dosing and Administration**	Extent to which the review discusses pharmacological protocols, dosing strategies, routes of administration, session procedures,	Dose range; session procedures; route of administration; monitoring protocols; frequency of	Discussion of psilocybin dosing protocols, ketamine administration models, session

Framework Domain	Operational Definition	Operational Focus	Examples of Explicit Coverage
	safety monitoring, or practical aspects of psychedelic administration.	administration; safety management; drug-session logistics.	monitoring procedures, or pharmacological safety considerations.

Table S4. Extended characteristics of included studies, including findings, safety, and methodological considerations.

Author, year	Review type	Psychedelics addressed	Population / Clinical context	Number of included studies	Outcomes analyzed	Main findings reported	Safety	Relevance to palliative care	Reported methodological limitations
**Maia et al., 2022 [11]**	Systematic review	"Lysergic acid diethylamide, psilocybin, and N,N-dipropyltryptamine"; also "ketamine" and "MDMA" in single studies.	"Patients diagnosed with life-limiting conditions, advanced or terminal illness"; predominantly "cancer patients."	"The sample was composed of 20 studies."	"The control of physical, psychological, social, and spiritual symptoms."	"reduced levels of anxiety and depression, reduced fear of death, and improvements in well-being, quality of life, and spirituality."	"The adverse effects reported were of physical and/or psychological nature and of mild to moderate intensity, transient, and self-resolutive"	"Therapeutic potential of psychedelic-assisted therapies for symptom control in patients with life-limiting conditions"	"Most studies have been conducted on cancer patients; therefore, the results may not be transferable to patients with different diagnoses"; "most of the studies were carried out in white and educated people"
**Belitzky et al., 2025 [16]**	"A Narrative Review"	"Psychedelics, such as lysergic acid diethylamide (LSD), psilocybin, mescaline, and N,N-dimethyltryptamine (DMT)."	"patients with cancer"; "In advanced cancer, pain is often anticipated to be persistent throughout the course of illness and end-of-life care."	"This search yielded 46 articles … which underwent evaluation."; and for trials, "Of the 23 included trials…"	"cancer pain and associated psychological distress"; "we use the term distress broadly to include cancer-related anxiety, depression, quality of life issues, and existential thoughts related to fear of death and dying or fear of cancer recurrence and progression."	"Early results are promising, and additional research is needed to understand efficacy and tolerability in broader cancer populations."	"While no serious adverse drug–drug interactions have been found, detailed medical screenings and further research … are needed."	"In advanced cancer, pain is often anticipated to be persistent throughout the course of illness and end-of-life care."	"Studies assessing psychedelics in patients with cancer for physical pain and psychological distress are case reports or small, open-label trials."

**Reiche et al., 2018 [17]**	"A systematic review"	"Lysergic acid diethylamide (LSD)"; "psilocybin"; and "dipropyltryptamine (DPT)."	"A systematic search for clinical trials from 1960 to 207 revealed 11 eligible clinical trials"	"symptoms of existential distress," including "anxiety and depression"; secondary reports included "quality of life and reduced fear of death.	"in palliative care"	"Evidence supports that patients with life-threatening diseases associated with symptoms of depression and anxiety enefit from the anxiolytic and antidepressant properties of serotonergic hallucinogens. "	"Low rates of side effects were reported in studies that adhered to safety guidelines"	"In palliative care, there is a growing consensus that existential distress is a core determinant of poor well-being and quality of life in patients with a life-threatening disease."	"Most of the studies before the year 2000 show strong methodological limitations"; "results were mainly based on anecdotal evidence or nonstandardized outcome criteria"; and "small sample size, cross-over design and inadequate blinding limit generalizability."
**Schuman, et al., 2025 [18]**	"A systematic review and meta-analysis"	"psilocybin"; "ketamine"; additionally "MDMA" and "LSD"	"adults with cancer or survivors" experiencing "psychosocial symptoms such as depression, anxiety, demoralization, and existential distress."	"Fifteen studies were included"	"psychosocial symptoms (e.g., depression, anxiety, suicidal ideation, existential distress)"	"Meta-analysis of four ketamine RCTs (n = 354) showed large, rapid effects on depression/anxiety"	"No treatment-related serious adverse events were reported across the psilocybin RCTs or non-randomized studies"	Ketamine showed "rapid-onset, clinically meaningful antidepressant effects within days of administration, even in late-stage palliative care"	"Overall certainty of evidence remains low" due to "small sample sizes, significant heterogeneity, imprecision, and methodological limitations"
**Marchi, et al., 2024 [19]**	"A systematic review and network meta-analysis"	"psilocybin"; "ketamine"; "3,4-methylenedioxymethamphetamine (MDMA)"; "lysergic acid diethylamide (LSD)."	"terminally ill people"; including "participants with cancer" and "other life-	"Nine studies, involving 606 participants were included"	"post-treatment measures of depression and anxiety, as proxies of existential distress"	"Network meta-analysis identified psilocybin as the most effective compound for depression,	"rates of treatment discontinuation and adverse events between psychedelics and controls were comparable"	"Psychedelics can be an effective and safe intervention in end-life care," addressing	"small number of RCTs," "difficulties in ensuring blinding procedures," "absence of direct comparisons between psychedelic compounds,"

			threatening conditions.”			and LSD for anxiety”		“existential distress”	
**Barbosa et al., 2023 [20]**	“a systematic review”	“ketamine”; including “racemic ketamine” and “S-ketamine (esketamine)”	“patients diagnosed with any life-threatening disease”	“32 studies” were included: “14 case reports, two case series, two quasi-experimental studies, and seven randomized clinical trials (RCTs)”	“depressive symptom outcomes,” including “changes in severity scale scores, response or remission rates, or subjective improvements in mood and behavior”	“Most case reports reported a robust effect”; however, “the larger studies reported conflicting findings.”	“Ketamine is generally safe”; across studies “there were no reports of serious adverse effects leading to treatment discontinuation”	“Although ketamine is generally safe and potentially useful, its efficacy in palliative care settings remains unclear”	“routes of administration, dosing regimens, and follow-up measures”
**Jing, Hoeh & Menkes, 2022 [9]**	“scoping review”	“Investigational drugs included ketamine (n = 11), psilocybin (n = 10), 3,4-methylenedioxy methamphetamine (n = 2), and lysergic acid diethylamide (n = 2)”	“with life-threatening illnesses or in palliative/hospice care” ; focus is “at end of life.”	“In total, 25 studies were eligible, including 13 randomized controlled trials and 12 open-label trials.”	“depression, anxiety, and existential distress at end of life.”	“A variety of ongoing or upcoming clinical trials are expected to usefully extend evidence regarding psychedelic-assisted group therapy and microdosing in the end-of-life setting.”	Not reported	“Modern palliative care calls for a multidimensional approach to improve quality of life, addressing physical, psychological, social, and spiritual or existential needs.”	“methodological challenges and sources of bias that beset psychedelic trials.”
**Schimmers et al., 2022 [21]**	“a systematic review”	“psilocybin”; “lysergic acid diethylamide (LSD)”; “3,4-	“patients with a terminal illness”; including	“33 studies were included”	“depression,” “anxiety,” and “existential distress”	“Most studies reported rapid and marked reductions in	“No serious adverse events were reported in	“psycho-existential distress in patients	“Small sample sizes,” “heterogeneity in study design and outcome measures,” and

		methylenedioxy methamphetamine (MDMA)"; "ketamine."	individuals with "advanced cancer" and other "life-threatening diseases."			depression and anxiety symptoms following psychedelic treatment"	the included studies"	facing terminal illness"	"difficulties maintaining blinding due to the psychoactive effects of psychedelics."
**Yu et al., 2021 [22]**	"A systematic review and meta-analysis"	"psilocybin."	"patients with life-threatening conditions, such as cancer and HIV"; focus on "end-of-life anxiety symptoms."	"Overall, five studies were included."	"changes in anxiety symptoms, including state anxiety and trait anxiety.	"Psilocybin was superior to the placebo in treating state anxiety at 1 day"	"Psilocybin was associated with transient elevation in systolic and diastolic (blood pressure compared with placebo."	Not reported	"small sample sizes"; "high heterogeneity on long-term outcomes";
**Schipper et al. 2024 [23]**	"Cochrane Database of Systematic Reviews – Intervention Review"	"classical psychedelics (psilocybin ('magic mushrooms') and lysergic acid diethylamide (LSD))" and "3,4-methylenedioxy methamphetamine (MDMA)."	"people with life-threatening diseases"	"We included six studies in the review … 149 participants were randomised and 140 analysed."	Anxiety (STAI-Trait; STAI-State), Depression (BDI; HADS-D), Existential distress (e.g., Demoralisation Scale).	Anxiety: "may result in a reduction in anxiety" compared to active placebo (low-certainty evidence).	"No treatment-related serious adverse events or adverse events grade 3/4 were reported."	The review states that anxiety, depression and existential distress are common and clinically important in people "receiving palliative care."	"Blinding could not be achieved as this is very difficult in studies investigating psychedelics."
**White et al., 2023 [24]**	"Comprehensive literature review"	"psilocybin, lysergic acid diethylamide (LSD), 3,4-methylenedioxy	"patients with cancer" and trials where patients "primarily had cancer	"Five unique randomized, double-blind, placebo-controlled trials"	Anxiety and depression measured using validated scales including "STAI-S, STAI-T, HAM-	"Significant reductions were found in 2 trials with 2 anxiety scales (State-Trait	"they had trained monitors or psychotherapists who supported them through their experience"	"These therapies may be promising for the treatment of	"The psychedelic experience is so prominent that it is difficult to devise a control situation that can mimic the experience to

		methamphetamine (MDMA)”	but could also have other life-threatening diseases”		A, HADS-A, HADS-D, BDI, GRID-HAM-D-17”	Anxiety Inventory–State, State-Trait Anxiety Inventory–Trait) and in 1 trial with 2 additional anxiety scales.”		feelings of anxiousness and depressed mood among patients with life-threatening cancer.”	effectively blind the patients, caregivers, and investigators.”
**Lapid et al., 2025 [25]**	“systematic review”	“psilocybin” / “psilocybin-assisted therapy”	Cancer patients with “psychological and existential distress” in “life-threatening cancer”	“Fourteen studies”	Depression, anxiety, existential distress; plus QoL / psycho-social-spiritual outcomes as reported across included studies	“consistently showed significant reductions in depression, anxiety, and existential distress, with improvements sustained over several months”	“Adverse effects were generally mild and transient”; RCTs reported “no serious adverse events”	distress is prevalent among patients with “life-threatening cancer” impacting QoL;	“relatively small sample sizes,” “modest study quality,” “open-label design… introduce potential biases”
**Stephen Ross, 2018 [26]**	“This article will systematically review the literature”	“psilocybin”, “lysergic acid diethylamide (LSD)”, and “dipropyltryptamine (DPT)”	“patients with cancer-related psychiatric distress”, “patients with advanced or terminal cancer diagnoses”	“The search found 10 eligible clinical trials”	“cancer-related depression, anxiety, fear of death, psychological and existential distress, quality-of-life, and spiritual well-being”	“Six open label trials, published between 1964 and 1980 (n = 341), suggested that psychedelic therapy (mostly with LSD) may improve cancer-related depression, anxiety, and fear of death”	“The safety of classic psychedelics (particularly psilocybin) in human research settings has been well documented when participants are carefully screened”	“treat cancer-related psychological and existential distress”	“The studies that have been completed to date are small open label or randomized controlled trials (RCTs)… and… are not sufficient to definitively establish treatment efficacy”

**Alexander et al., 2025 [27]**	"narrative review"	"psilocybin" and "lysergic acid diethylamide (LSD)"	"palliative care", "patients with an advanced illness", and "patients with life-threatening illness"	Not reported	"meaning", "existential distress", "demoralization", "quality of life", "anxiety", "depression", and "suicide risk"	"early clinical trial data indicate that psychedelic therapies show promise for existential distress, including demoralization"	"transient paranoia and delusional thoughts are reported in a small proportion of patients"	"psychedelic therapy is emerging as a promising therapeutic approach in palliative care"	"early clinical trial data" and need for "further investigation"
**Moshfeghinia et al., 2026 [28]**	"A systematic review and meta-analysis"	"Psilocybin"	"cancer patients" / "Adults aged 18 and older diagnosed with cancer."	"eight studies … being included in the final analysis."	"anxiety, depression, and other mental outcomes" and "psychological well-being, depression, anxiety, spiritual well-being, and quality of life"	"In randomized controlled trials (RCTs), psilocybin significantly reduced depressive symptoms, with the Beck Depression Inventory (BDI) (standardized mean difference [SMD] = −2.87, 95% confidence interval [CI]: −3.99 to −1.76, $p < 0.001$)"	"Adverse effects were generally mild and transient."	"Not reported"	"Given the small number of studies, high heterogeneity, challenges with blinding/expectancy, and frequent co-intervention with psychotherapy, these findings are preliminary."
**Ferreira et al, 2025 [29]**	"systematic review"	"psilocybin", "3,4-Methylenedioxy methamphetamine (MDMA)", "lysergic acid	"patients with life-threatening, incurable, or terminal illnesses" and	"six studies"	"spirituality", "spiritual well-being (SpWB)", and "mystical experience and its relationship	"Psychedelic-Assisted Therapy (PAT), especially with psilocybin, demonstrated	"no serious adverse events were reported"	"the potential of PAT to address unmet spiritual	"the small sample size, reflected in the limited number of studies, each of which individually has a small sample size"

		diethylamide (LSD)", "mescaline", "dipropyltryptamine	"palliative care (PALC)"		with spirituality-related outcomes"	significant enhancements in spirituality, mystical experiences, and SpWB"		needs and enhance SpWB in patients with life-threatening illnesses"	
**Bader et al., 2024 [30]**	"Systematic review and meta-analysis"	"psilocybin"	"adult patients with advanced cancer"	"7 studies"	"quality of life, pain control, and anxiety relief"	"significant improvements in quality of life, pain control, and anxiety relief following psilocybin-assisted therapy"	"No significant adverse events related to psilocybin were reported"	"highlight the potential therapeutic benefits of psilocybin in palliative care settings"	"heterogeneity in symptom reporting and study structures precluded achieving consistent homogeneity necessary for conducting a meta-analysis across all these symptoms"
**Ko et al., 2022 [31]**	"Systematic Review"	"psychedelic therapy utilizing psilocybin, ayahuasca, or ketamine"	"adult subjects with psychiatric and/or addictive disorders who received psychedelic dosing either in laboratory or clinical setting"	12 studies	"association between mystical experience and symptom reduction"	"Ten of the twelve established a significant association of correlation, mediation, and/or prediction."	"To date, studies indicate not only effectiveness but also safety, with fewer potential side effects than other forms of medication"	Not reported	"A majority of the studies are limited, however, by their small sample size and lack of diversity (gender, ethnic, racial, educational, and socioeconomic)"
**Kratina et al., 2026 [32]**	"A scoping review"	"ketamine; psilocybin; ayahuasca; MDMA; N,N-Dipropyltryptamine (DPT); LSD; ibogaine; peyote; and mescaline"	"populations coping with psychological suffering associated with life-threatening illness and the	"Fifty-nine studies on six types of psychedelic substances for end-of-life issues were identified"	"Outcome measures spanned biopsychosocial-spiritual domains"	"affective and cognitive-affective sub-domains most often assessed"	"Most studies reported challenging experiences, with a large proportion considering them therapeutic."	Not reported	"The heterogeneity in types of TPIs, study designs, methodologies, and outcome measures across the included studies could have led to overly generalized findings."

			end of life itself”						
**Amaev et al., 2025 [33]**	“A systematic review and meta-analysis”	“Serotonergic psychedelic assisted therapy (PT)” and “psilocybin”, “LSD”, “MDMA”	“DA may underpin IA and is more common in individuals with life-threatening illness”	“Five randomized controlled trials were included.”	“death anxiety (DA)”, “illness anxiety (IA)”, and “State Trait Anxiety Inventory (STAI) State and Trait scales”	“The results showed that PT was associated with sustained decreases in DA and State and Trait anxiety in the context of having a life-threatening illness.”	“Not reported”	“anxiety associated with life-threatening illness”	“high risk of bias for blinding of participants, personnel, and outcome assessment was high for all studies due to limited blinding efficacy”
**Thivya Turner and Paul Glue 2025 [34]**	“Systematic Review”	“psilocybin, two studies used MDMA, two studies used LSD, and one study used dipropyltryptamine (DPT).”	“patients with life-threatening illness” and “individuals with a physical life-threatening illness with associated distress, depression, and/or anxiety symptoms”	“A total of 14 studies met the inclusion criteria” and “A total of 14 articles met the inclusion criteria”	“outcome measures related to attitudes toward life, death, and spirituality”	“Improvements in distress, depression, and/or anxiety symptoms were associated with reduced demoralization, reduced hopelessness, improvements in spirituality”	“No deterioration was reported in any measures of attitudes toward life, death, and spirituality	“life-threatening illness”, “end of life”	“Results need to be considered with caution, given the paucity and quality of available data.”
**Sholevar et al., 2025 [35]**	“A Narrative Review”	“Ketamine” and “ketamine-assisted psychotherapy (KAP)”	“patients with serious medical illness” and “palliative care setting”	“Nine studies and 12 case reports were identified”	“psychiatric and existential distress” and examples “demoralization, death anxiety, spiritual distress”	“Evidence suggests that ketamine may induce rapid and transient improvements in psychiatric symptoms in patients with	“reported positive results and a good safety profile” and “There were no serious adverse events reported across	“psychiatric and existential distress… frequently encountered in the palliative care setting”	“Our review was not a formal systematic review and, therefore, may be limited in its comprehensiveness.”

						serious medical illness.”	all case reports and studies.”		

Table S5. Overview of framework domains addressed in the included reviews of psychedelic-assisted interventions in palliative care.

Author, year	Length of therapy	Important Indications	Intrinsic Motivation	Mystical-type experiences	Integration with PC Healthcare Model	Personal or family issues	Clinical Training	Biographical and Relational Questions	Dosing and administration
**Maia et al., 2022 [11]**	"Preparatory psychotherapy (1–2 hours nondrug sessions)" "Drug (psychedelic) dosing sessions (6 –14 hours)" "Post-dosing integrative psychotherapy (1–2 hours nondrug sessions)"	"symptom control in patients diagnosed with serious illness" "The control of physical, psychological, social, and spiritual symptoms" (exemplos citados nos resultados): "reduced levels of anxiety", "depression", "fear of death", "improved spiritual well-being"	Absent	"The most dramatic effects occurred following the mystical psychedelic experience" "The mystical experience induced by psilocybin mediated the therapeutic effect of this substance on anxiety and depression."	"patients with life-limiting conditions, advanced or terminal life-threatening illness"	"to review the participants’ life histories" ; "to review the nature and status of present relationships and concerns"	"the therapist's training in this specific psychotherapeutic intervention" "Therapists need to be properly trained to work in the field of psychedelic-assisted therapy."	"to review the participants’ life histories" ; "to review the nature and status of present relationships and concerns"	"The routes of administration used were oral (n = 8, 40%)" "intramuscular (n = 4, 20%)" "subcutaneous for LSD (n = 1, 5%)" "In 5 (25%) studies, no information was provided regarding the administration route used."
**Belitzky et al., 2025 [16]**	"group sessions both in preparation and as a follow-up"	"cancer pain and associated psychological distress" "Distress ... include cancer-related anxiety, depression,	Absent	"The mystical experience induced by psilocybin mediated the therapeutic effect of this substance on	"Psilocybin combined with multidisciplinary palliative care in demoralized cancer	Absent	"Research on psychedelics should also provide specifics of the nature of psychotherapy such as	Absent	"The threshold oral dose of LSD is between 20 and 30 µg."; "Psilocybin is less potent, requiring oral doses between 4 and 10 mg to have an effect."; "Depending on dosage,

		quality of life issues, and existential thoughts related to fear of death and dying or fear of cancer recurrence and progression."		anxiety and depression."	survivors with chronic pain"		theoretical orientation, background of the interventionalist, number and length of sessions, and fidelity to the protocol."		the effects can persist for approximately 6 h"
**Reiche et al., 2018 [17]**	Absent	"treatment of anxiety and depression in patients suffering from life-threatening diseases" ; "symptoms of existential distress"	Absent	"mystical-type experiences"; "Degree of positive change correlates with the occurrence of 'mystical' experiences"	"in palliative care"	"daily interviews were held for one week to establish a trustful relationship with the patients and discuss relevant personal issues"	Absent	"The patient's biographical and medical history, their intention to participate, treatment goals, and the structure of the sessions were discussed prior to the sessions"	"V: 0.2 mg/kg (moderate dose) P: Niacin (250 mg)"; "V: 22 or 30 mg/70 kg (high dose)" e "AP: 1 or 3 mg/70 kg (very low dose)"
**Schuman, et al., 2025 [18]**	Absent	"psychological distress like anxiety, depression, and existential suffering in adults with cancer" ; "psychosocial symptoms (e.g., depression, anxiety, suicidal ideation, existential distress)"	Absent	"mystical-type experiences" ; "therapeutic outcomes were often correlated with the intensity of mystical-type experiences"	"advanced cancer"; "palliative care"	Absent	"trained therapists"; "structured psychological support"	"meaning making"; "existential distress"	Psilocybin: "25 mg"; "0.2 mg/kg"; "0.3 mg/kg" Ketamine / Esketamine: "0.1–0.5 mg/kg IV"; "intranasal ketamine (50–150 mg)"

**Marchi, et al., 2024 [19]**	"The median duration of the trials was 28 days, ranging from 3 days to 6 months."	"Post-treatment measures of depression and anxiety, as proxies of existential distress, and tolerability were the primary outcomes."	Absent	"mystical-type experiences"; "a heightened sense of introspection, and mystical-type experiences"	"palliative care" and "end-of-life settings"	Absent	Absent	"loss of meaning, death anxiety, and despair"; "spirituality, empathy, introspection, and relaxation of high-level beliefs"	Dosing and administration "0.2 mg/kg psilocybin po"; "250 mg niacin po" "0.3–0.4 mg/kg psilocybin po" e "0.01–0.04 mg/kg psilocybin po" "0.5 mg/kg racemic ketamine iv" e "0.05 mg/kg midazolam iv" "0.125 mg/kg racemic ketamine iv" "200 µg LSD po" e "20 µg LSD po" "125 mg MDMA po" e "125 mg lactose po"
**Barbosa et al., 2023 [20]**	"with any treatment/follow-up duration"	"ketamine for the treatment of depressive symptoms in palliative care"; "depression treatment within a broad palliative care concept"	Absent	Absent	"within a broad concept of palliative care according to the World Health Organization (WHO) definition" "interventions promoting the quality of life of people suffering from life-threatening illnesses"	Absent	Absent	Absent	"via any route of administration"; "a single 0.5 mg/kg IV ketamine infusion" "daily oral ketamine doses for 28 days (up to 0.5 mg/kg per dose)" "a single dose of 0.25 mg/kg IV esketamine" "intranasal ketamine doses (50-150 mg) at 3-day intervals" (perioperatório): "0.5 mg/kg IV racemic ketamine"; "0.5 mg/kg (high-dose) S-ketamine"; "0.25 mg/kg (low-dose) S-ketamine"
**Jing, Hoeh & Menkes, 2022 [9]**	Absent	"depression, anxiety, and existential distress at end of life"	Absent	"mystical-type experience, defined by feelings of unity,	"Modern palliative care calls for a multidimensional approach to	Absent	Absent	Absent	Psilocybin: "single doses of either 25 mg or 1 mg (active control) of psilocybin"

				interconnectedness, peace, and joy, as well as senses of sacredness, ineffability, and transcendence"	improve quality of life, addressing physical, psychological, social, and spiritual or existential needs"				Microdosing: "psilocybin microdosing (1–3 mg/day, Monday–Friday for 4 weeks)"<br>LSD microdosing: "titrated dose of LSD (4–12 μg) … twice a week for 6 weeks"<br>NIMH trial: "20 mg psilocybin, 200 mg ketamine, and 5 mg midazolam"<br>MDMA: "either 120 mg MDMA or 20 mg methylphenidate"
**Schimmers et al., 2022 [21]**	Absent	"existential distress, depression, and anxiety"	Absent	"the occurrence of a 'psychedelic peak experience' seemed positively correlated with therapeutic effect"	"psychedelic treatment may be a suitable option in palliative care given the importance that is placed upon existential and spiritual themes."	"closeness of family relationships"; "Both participants and their friends and family reported positive effects"	Absent	"the therapist got to know the patient, rapport was established, and the patient was prepared for the procedure"	"DPT, 75–127.5 mg, IM"<br>LSD: "LSD, 200–400 μg, PO, IV, or IM"<br>Psilocybin: "psilocybin (0.2 mg/kg) or niacin (250 mg)"<br>Psilocybin (Griffiths/Ross): "22–30 mg/70 kg" e "0.3 mg/kg"<br>Ketamine (Irwin): "28 continuous day administrations of oral ketamine (0.5 mg/kg)"<br>Ketamine (Iglewicz): "ketamine (0.5 mg/kg) … administered orally … subcutaneously"<br>MDMA: "MDMA, 125 mg … optionally followed by 62.5 mg MDMA-dose"
**Yu et al., 2021 [22]**	"oral single dose"; "day 1 and month 1,	"end-of-life anxiety symptoms";	Absent	"Psilocybin is reported to produce highly	"in the palliative care and psycho-	Absent	Absent	Absent	"Oral single dose, 0.20 mg/kg"; "Oral single dose, 0.32 mg/kg (22 or

	3, and 6 after single session of psilocybin treatment"	(outcomes): "state anxiety" e "trait anxiety"		spiritual states of consciousness"	oncology disciplines, psilocybin can be considered a treatment option under controlled clinical practice for patients with cancer or HIV"				30 mg)" ; "Oral single dose, 0.3 mg/kg" "Oral single dose, 0.30–0.36 mg/kg"
**Schipper et al. 2024 [23]**	"The studies lasted between 6 and 12 months"; "one or two psychedelic-assisted sessions accompanied by preparation and integration therapy sessions"	"anxiety" "depression" "existential distress" "demoralisation"	Absent	"Participants receiving psychedelic-assisted therapy with classical psychedelics rated their experience as being spiritually significant"	"people with life-threatening diseases"	Absent	"psychedelic-assisted therapy refers to a group of therapeutic practices involving psychedelics taken under therapeutic supervision from physicians, psychologists, and others"	"existential distress" and "demoralisation"	Psilocybin: "0.2 to 0.4 mg/kg" ou "25 mg" LSD: "200 µg" MDMA: "75 to 125 mg"
**White et al., 2023 [24]**	"Only a few sessions may also provide benefits extending out for 6 to 12 months and possibly beyond that."	"the treatment of anxiety or depression among patients with cancer"	Absent	"Psilocybin provided a psychedelic effect in this trial as evidenced by the Mystical Experience Questionnaire	"7%-49% in palliative care"; "we found that the patients were seldom in the terminal stages of their disease"	"patients ... better understood or appreciated the struggles of family members"; "several patients felt they reached out more to family"	"they had trained monitors or psychotherapists who supported them through their experience"	"allowed them to see their lives and their disease in a more objective way"; "connection to the wholeness of nature and life's plan" e "reconciliation about the	"LSD 200-500 µg ± psychotherapy"; "LSD 200 µg ... Placebo (LSD 20 µg)"; "Psilocybin 0.2 mg/kg"; "Placebo (niacin 250 mg)"; Psilocybin 22-30 mg (~0.3-0.4 mg/kg)"; "Placebo (psilocybin 1-3 mg)"; "Psilocybin 0.3 mg/kg ± psychotherapy" e "Placebo (niacin 250 mg) ± psychotherapy";

								thought of experiencing death"	
**Lapid et al., 2025 [25]**	"improvements sustained over several months"	"reducing psychological and existential distress in cancer patients"; "depression, anxiety, and existential distress"	Absent	"the influence of mystical experiences"; "ego dissolution"; "ineffability, transcendence of time and space, universal interconnectedness, and a deeply felt positive mood"	"particularly relevant in palliative care settings"	"cancer distress can profoundly impact both patients and their families' quality of life"	"comprehensive support from trained therapists"; "Integrating psychedelic therapy education into psychiatric and clinical psychology training programs"	"discussion of meaningful aspects of participant's life"; "Discussion of meaningful life aspects and preparation for psilocybin experience."	"Single high dose 22 mg/70 kg"; "Single low dose 3 mg/70 kg ... 1 mg/70 kg" ; "Single moderate dose (0.2 mg/kg)" e "Single dose niacin (250 mg)"; "Single moderate dose (0.3 mg/kg)";"Single dose niacin (250 mg)"; "Single dose 25 mg"; "Single dose 0.3 mg/kg ... 0.36 mg/kg"
**Stephen Ross, 2018 [26]**	Absent	"cancer-related depression, anxiety, and fear of death"; "psychological and existential distress"	Absent	"reports of mystical-type experiences ('oceanic feelings')"; "positive correlation between the occurrence of the 'mystical experience' and clinical improvements"	"advanced or terminal cancer"; "Use of psilocybin-assisted treatment in the terminally ill could be especially useful for patients in inpatient or outpatient hospice settings"	Absent	"Training of therapists and fidelity to treatment models would have to be considered, and it would be important to establish specific psychedelic-assisted therapy training programmes."	"after a thorough life review and review of their cancer diagnosis and its negative psychological and existential impact"	"10 eligible clinical trials, with a total of 445 participants"; "LSD (n=323)", "psilocybin (n=92)", "DPT (n=30)"; "LSD (100mcg orally)"; "high-dose oral LSD (200–500mcg orally)"
**Alexander et al., 2025 [27]**	"preparation" + "dosing session" + "integration"	"Loss of meaning contributes to existential distress, and, in particular, may manifest as	Absent	"elicits a mystical-type state"	"palliative care setting"	Absent	"the need for this is increasingly recognized"	"meaning-making... the restoration of meaning in the context of	"moderate-to-high dose of a psychedelic compound"

		demoralization"; "demoralization... linked with diminished quality of life, increased symptom burden, and increased suicide risk"						highly stressful situations"	
**Moshfeghinia et al., 2026 [28]**	"short-term (2–5 weeks) and long-term (6 months) follow-ups"	"psychological well-being, depression, anxiety, spiritual well-being, and quality of life".	Absent	"Classical psychedelics can also cause mystical experiences, which encompass such features as ineffability, transcendence of time and space, universal interconnectedness..."	“psychological distress in cancer patients”, e discute “spiritual well-being”	Absent	"underscoring the importance of safety guidelines and clinical supervision"; "psilocybin is functional only when attentively considering the conduct of set and setting"	"loss of spirituality is another important dimension of psychological distress, defined as a disruption in one’s sense of life purpose, connection to others, and relationship with a higher power"	"0.3 mg/kg" (Ross 2016 e follow-ups) ; "between 0.31 and 0.43 mg/kg" (Griffiths 2016 estimado) "25 mg" (Lewis 2023; Shnayder 2023; Agrawal 2023); "0.2 mg/kg" (Grob 2011)
**Ferreira et al, 2025 [29]**	"two day-long experimental sessions scheduled two to four weeks apart"; "one oral dosing session"	"spirituality"; "spiritual well-being (SpWB)"; "mystical experiences"	"spirituality can be comprehensively described as a dynamic and intrinsic aspect of humanity through which individuals seek ultimate meaning, purpose, and	"mystical experiences are commonly reported with the use of psychedelic substances"	"Within palliative care (PALC)"; "ensure safe and effective implementation in palliative care settings"	"experience a relationship with themselves, family, others, community, society, nature, and the significant or sacred"	"rigorous evaluation of healthcare practitioners’ role in guiding patients through PAT protocols is essential"	"seek ultimate meaning, purpose, and transcendence"; "relationship with themselves, family, others, community"; "personally meaningful insights"	"0.3 mg/kg psilocybin"; "22 or 30 mg/70 kg" "25 mg psilocybin session"; "two day-long 125 mg MDMA experimental sessions"

			transcendence"						
**Bader et al., 2024 [30]**	Absent	"Reporting outcomes related to quality of life, pain control, or anxiety relief."	Absent	"a single session of psilocybin induced a mystical encounter for the patient."	"potential therapeutic benefits of psilocybin in palliative care settings."	Absent	Absent	"meaning-making"; "Participants attributed their improved attitudes towards self, mood, life, personal relationships, and spirituality to their experiences"	"Psilocybin (0.3 mg/kg) on the first medication session followed by niacin (250 mg) on the second session"
**Ko et al., 2022 [31]**	Absent	"cancer-related distress, substance use disorder, and depressive disorders"; "symptom reduction and improved quality of life"	Absent	"The mystical experience is a potential psychological mechanism to influence outcome in psychedelic therapy"	"cancer-related distress"	Absent	Absent	"improved quality of life"; "personally meaningful insight"	"psilocybin doses ranging from 10 to 30 mg dependent on body mass"
**Kratina et al., 2026 [32]**	"the number of sessions ranged from a single follow-up, daily meetings for three weeks, to 6 h over six weeks"	"psychological suffering associated with confronting end-of-life issues"; "existential distress, death anxiety, hopelessness, perceived loss of dignity, and diminished	"distress arising from coping with a foreshortened future in response to a life-threatening illness"	"spiritual measures of transcendental experiences, e.g. dissolution of ego boundaries, sense of connectedness to the universe"	"palliative care clinicians"; "therapeutic psychedelic interventions in the context of end-of-life care"	"early studies … involved family members in preparatory work"; "family members in integration"	"qualifications of the therapist or guide"	"participants discussed various aspects of their lives"; "life review"	.5 mg/kg" (ketamine IV) "22 mg/70 kg"; "30 mg/70 kg" (psilocybin); "100 mcg to 500 mcg" (LSD); "125 mg" (MDMA)

		spiritual well-being"							
**Amaev et al., 2025 [33]**	"two 200 µg doses of LSD"; "two doses of 125 mg of MDMA"; "two 200 µg doses of LSD … separated by 6 weeks"	"death anxiety (DA)"; "illness anxiety (IA)"; "anxiety in the context of a life-threatening illness"	"preoccupation with having or acquiring a serious physical illness (typically a life-threatening disease)"	Absent	"life-threatening illness"; "terminal illness"	Absent	Absent	Absent	"22 or 30 mg/70 kg" (psilocybin) "0.3 mg/kg" (psilocybin) "200 µg" (LSD) "125 mg" (MDMA)
**Thivya Turner and Paul Glue 2025 [34]**	"preparation therapy sessions"; "psychedelic-assisted therapy session"; "integration therapy sessions"	"distress, depression, and/or anxiety symptoms in life-threatening illness"	"greater sense of meaningful existence"; "greater sense of optimism toward life"	"mysticism subscale"; "ineffability"; "from separateness to interconnectedness"	"palliative care context"; "end of life context"	"Families were also seen with and without the patient"; "Family involved in latter part of session"	"non-directive, supportive therapeutic approach"; "preparation therapy sessions"	"revised life priorities"; "wisdom lessons"; "reconnection to life"; "reconciliation with life and death"	22 mg/70 kg or 30 mg/70 kg oral psilocybin" "0.3 mg/kg oral psilocybin" "125 mg oral MDMA" "200–500 mcg oral LSD" "75–127.5 mg intravenous DPT"
**Sholevar et al., 2025 [35]**	"single dose"; "multiple doses … over periods of time as long as 36 weeks"; "daily PO ketamine … for periods of time ranging from 9 to 136 days"	psychiatric and existential distress"; "depression"; "anxiety"; "demoralization" "death anxiety"; "spiritual distress"	"existential distress"; "loss of autonomy"; "demoralization"	"mystical-type experiences"; "ego-dissolving transcendental experiences"; "out-of-body experiences (OBEs)"; "near-death experiences (NDEs)"	palliative care setting"; "hospice"; "inpatient palliative care unit"	"families were also seen with and without the patient"	"interdisciplinary team"; "psychiatric consultation"	"anticipatory grief resolution (AGR)"; "wish to hasten death"	"intravenous (IV), intramuscular (IM), intranasal (IN), subcutaneous (SC), sublingual (SL), and oral (PO)" "0.5 mg/kg" "150 mg IM" "25–400 mg SL"

PC, palliative care; PAT, psychedelic-assisted therapy; PAP, psychedelic-assisted psychotherapy; KAP, ketamine-assisted psychotherapy; EOL, end of life; EOLD, end-of-life distress; QoL, quality of life; DA, death anxiety; IA, illness anxiety; LSD, lysergic acid diethylamide; DMT, N,N-dimethyltryptamine; MDMA, 3,4-methylenedioxymethamphetamine; MEQ-30, Mystical Experience Questionnaire–30 item; GOCD, goals of care discussion; IV, intravenous; IM, intramuscular; IN, intranasal; PO, oral administration; SC, subcutaneous.
